# Primary cilium loss in mammalian cells occurs predominantly by whole-cilium shedding

**DOI:** 10.1371/journal.pbio.3000381

**Published:** 2019-07-17

**Authors:** Mary Mirvis, Kathleen A. Siemers, W. James Nelson, Tim P. Stearns

**Affiliations:** 1 Department of Molecular and Cellular Physiology, Stanford University, Stanford, California, United States of America; 2 Department of Biology, Stanford University, Stanford, California, United States of America; 3 Department of Genetics, Stanford University, Stanford, California, United States of America; Institut Curie, FRANCE

## Abstract

The primary cilium is a central signaling hub in cell proliferation and differentiation and is built and disassembled every cell cycle in many animal cells. Disassembly is critically important, as misregulation or delay of cilia loss leads to cell cycle defects. The physical means by which cilia are lost are poorly understood but are thought to involve resorption of ciliary components into the cell body. To investigate cilium loss in mammalian cells, we used live-cell imaging to comprehensively characterize individual events. The predominant mode of cilium loss was rapid deciliation, in which the membrane and axoneme of the cilium was shed from the cell. Gradual resorption was also observed, as well as events in which a period of gradual resorption was followed by rapid deciliation. Deciliation resulted in intact shed cilia that could be recovered from culture medium and contained both membrane and axoneme proteins. We modulated levels of katanin and intracellular calcium, two putative regulators of deciliation, and found that excess katanin promotes cilia loss by deciliation, independently of calcium. Together, these results suggest that mammalian ciliary loss involves a tunable decision between deciliation and resorption.

## Abstract 2

Disassembly of the primary cilium is required for cell cycle progression. Live cell imaging reveals that this process involves a tunable decision between gradual resorption and rapid deciliation; in IMCD3 mammalian kidney cells, the predominant behavior is deciliation.

## Introduction

Cilia are present in all branches of the eukarya. These organelles are adapted to generate motion and facilitate interactions between cells and their surrounding environment through the transduction of molecular and mechanical signals [1–3]. All cilia share a core architecture consisting of a basal body (centriole), an axoneme of stable microtubule doublets, and a ciliary membrane [4–6]. In many cases, including vertebrate primary cilia and *Chlamydomonas* motile cilia, the cilium is closely linked with cell cycle stage, generally forming in interphase (G0) and disassembling at some point prior to mitosis [6–12]. Misregulation or delay in primary cilia loss leads to defects in cell cycle progression, which underlie aberrant developmental and homeostatic phenotypes in ciliopathies and many cancers [7,13–26].

Despite recent progress in the identification of molecular players and pathways regulating primary cilia disassembly [12,17, 19, 27–36], the physical mechanisms by which mammalian cilia are ultimately lost have not been identified. In *Chlamydomonas*, cilia are resorbed into the cell body preceding cell division with rates of approximately 0.3–0.7 μm/min [21,29, 32–34,37–45]. Alternatively, conditions of stress or pharmacological induction have been shown to trigger comparatively rapid removal of cilia, defined as the concurrent release of membrane and axoneme from the cell body. This phenomenon has been described in many species as deciliation, ciliary excision, shedding, deflagellation, or flagellar autotomy [46–53]. Whether deciliation occurs as part of normal primary cilium behavior and contributes to cell cycle–linked cilia loss is unknown. In this work, we use long-term live-cell imaging to determine how these physical mechanisms (resorption or deciliation) contribute to mammalian cell cycle–associated cilia loss.

## Results

### Ciliary structures in cells undergoing serum-induced cilium loss

**Fig 1:**
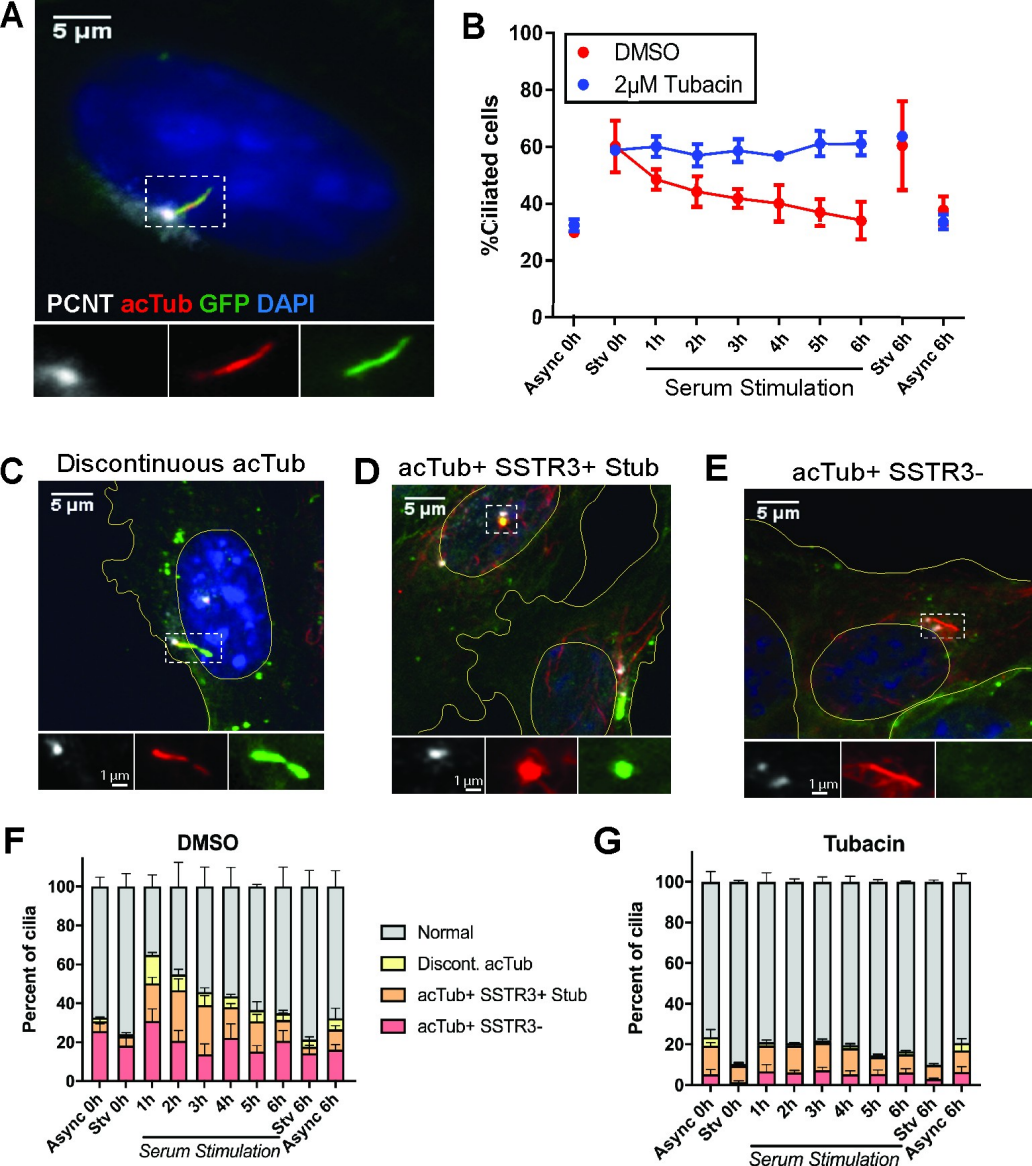
Serum stimulation of IMCD3 cells reveals noncanonical ciliary structures. IMCD3-SSTR3::GFP cells were serum starved for 24 hrs, followed by stimulation with 10% serum in order to synchronize ciliary loss. A, C–E) Cells were fixed at indicated time points after serum addition and immunostained for PCNT to mark the basal body (white) and acTub to mark the axoneme (red). Nuclei and cell boundaries are outlined in yellow. A) Morphology of a normal, intact cilium in a starved cell. B) The population of ciliated cells quantified over a serum-stimulation time course. Async and serum-Stv controls were included at 0 hrs and 6 hrs. Cells treated in parallel with 2 μM tubacin. C–E) Noncanonical ciliary structures identified in serum-stimulated cell populations. C) Discontinuous acTub staining, in this case accompanied by narrowing of the membrane. D) A ciliary stub, marked by punctate acTub and SSTR3 fluorescence. E) Full-length axoneme marked by acTub, lacking corresponding SSTR3::GFP signal. F–G) Stacked plots of cilia morphologies observed during serum stimulation in (F) DMSO and (G) tubacin-treated cells. Quantifications are based on means of three independent experiments with 150–200 cells analyzed per condition per replicate. Error bars = SEM. Source data can be found in supporting data file S1 Data. acTub, acetylated tubulin; Async, asynchronous; IMCD3; PCNT, pericentrin; SSTR3::GFP, somatostatin receptor 3::green fluorescent protein; Stv, starved.

To assess the physical processes underlying ciliary loss, we observed ciliary morphology in a population of mammalian cells entering the cell cycle. We manipulated serum level in the culture medium to synchronize ciliary behavior in inner medullary collecting duct 3 (IMCD3) cells expressing somatostatin receptor 3::green fluorescent protein (SSTR3::GFP) [24,54,55], a fluorescent plasma membrane marker that is enriched in the ciliary membrane (Fig 1A and S1A Fig). The majority of serum-starved cells (60 +/− 9.09%) were ciliated. Subsequent serum stimulation for 6 hrs resulted in a decrease in the fraction of cells with a cilium (30 +/− 0.4%) to levels comparable to asynchronously cycling cells. Serum-induced cilium loss requires the function of histone deacetylase 6 (HDAC6), a deacetylase of tubulin and cortactin [24,34]. Cells treated with 2 μM tubacin, an HDAC6 inhibitor, failed to undergo serum-induced ciliary loss (Fig 1B) [24,56,57]. Mitotic cells accumulated following serum stimulation, but this accumulation was inhibited or delayed in tubacin-treated cells (S1B Fig), consistent with a requirement for ciliary loss prior to mitotic entry [10,11,58–60]. Thus, serum stimulation induces semisynchronous, HDAC6-mediated, cell cycle–linked ciliary loss in these cells.

**Fig 2:**
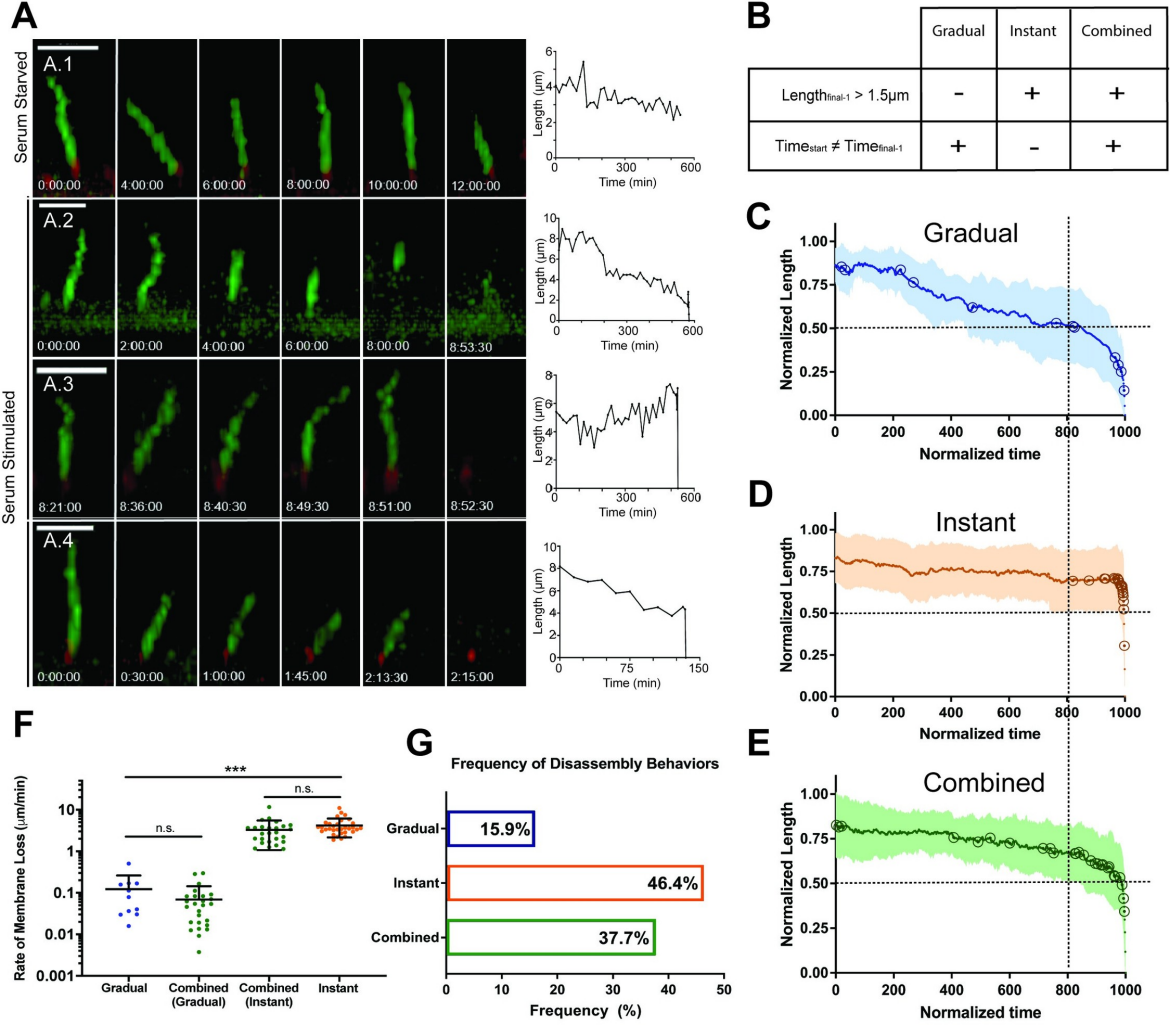
Live-cell analysis of ciliary loss reveal highly heterogeneous dynamics. A) Still images and raw length curves representing dynamics of individual cilia-loss events. Length measurements based on SSTR3::GFP fluorescence. Scale bar = 5 μm. A.1) Control serum starved cilium measured over a 12-hr period undergoes slight length change of 1.66 μm at a rate of 0.003 μm/min. A.2) Gradual loss with a rate of 0.016 μm/min. A.3) Instant loss, with an approximate minimum rate of 4.72 μm/min. A.4) Combined loss, consisting of an initial stage of Gradual shortening at a rate of 0.029 μm/min, followed by Instant loss at a minimum rate of 5.69 μm/min. B–E) Semiautomated analysis of loss events. B) Criteria for defining Gradual, Instant, and Combined behaviors. See text and S2 Fig for more details. C–E) Cumulative normalized length versus time curves for all (C) Gradual, (D) Instant, and (E) Combined events. Open circles indicate individual event start points. Dashed lines at 0.5 normalized length and 800 normalized time units to highlight differences in dynamics. F) Cilia-loss rates within dynamic groups (Gradual *n* = 11, Instant *n* = 32, Combined *n* = 26). Significance was determined by unpaired *t* test, *P < 0.05, **P < 0.01, ***P < 0.001. Error bars = SD. H) Relative frequency of behaviors (*n* = 69). Source data can be found in supporting data file S2 Data. SSTR3::GFP, somatostatin receptor 3::green fluorescent protein.

We next characterized cilia morphology in fixed cells over a 6-hr serum-stimulation time course. We identified three major types of noncanonical ciliary structures as likely loss intermediates in fixed cells expressing SSTR3::GFP (Fig 1C–1G): 1) a discontinuous axoneme, marked by a gap in acetylated tubulin (acTub) staining in all z-planes (“Discontinuous acTub,” Fig 1C); 2) a ciliary stub, characterized by a short (<1 μm) cilium positive for both acTub and SSTR3 membrane fluorescence (“acTub+ SSTR3+ Stub,” Fig 1D); and 3) an axoneme marked by linear acTub fluorescence (>1 μm) but lacking corresponding membrane SSTR3 fluorescence (“acTub+ SSTR3−,” Fig 1E). All intermediate structures were rare in serum-starved cells but at 1–2 hr after serum stimulation comprised the majority of detectable cilia (Fig 1F). Tubacin treatment prevented the enrichment of all three structures in serum-stimulated cells (Fig 1G), indicating that these structures are most likely representative of disassembling cilia.

### Rapid deciliation is the predominant mode of ciliary loss

These results suggested that ciliary-loss behaviors within a single-cell population might be variable, so we used live-cell imaging to observe individual cilia-loss events. We generated IMCD3-SSTR3::GFP cells that stably coexpress a centrosome marker (mCherry-PACT) to identify the position of the basal body. Live cells were imaged immediately following serum stimulation, with full confocal stacks acquired continuously for 6–12 hrs at 90-sec intervals (see Materials and methods). Apically-facing cilia were selected for analysis to avoid artifacts caused by deformations of basally-facing cilia by interactions with the culture substrate. Control serum-starved cells subjected to this imaging regime retained their cilia over 6 hrs (S2A Fig) and exhibited only a slight decrease in length over 12 hrs (S2B Fig), suggesting that imaging itself does not stimulate cilium-loss dynamics. The mean standard deviation of variation between consecutive length measurements was 1.53 μm, likely reflecting measurement error intrinsic to 3D visualization with our spatiotemporal resolution (S2B Fig). These metrics in serum-starved cells were used as baseline controls for serum-induced ciliary loss (Fig 2A.1, S2A and S2B Fig).

Image sequences of cells undergoing cilia loss revealed a striking range of dynamic behaviors (Fig 2A, S1 Movie, S2 Movie, S3 Movie and S4 Movie). We grouped these behaviors into three categories: Gradual, cilium length reduction over at least two consecutive time points resulting in terminal cilium loss (e.g., Fig 2A.2; 0.02 μm/min; T_start_ ≠ T_final-1_; see S2 Fig); Instant, a single discrete cilium-loss event within a single-imaging frame, i.e., 30–90 sec (e.g., Fig 2A.3; ≥4.72 μm/min L_final-1_ > 1.5 μm); and Combined, a period of Gradual disassembly directly followed by Instant loss (e.g., Fig 2A.4; Gradual phase [0.03 μm/min], preceding rapid loss within 46 seconds, ≥5.69 μm/min). To reduce bias in our cumulative analysis, we developed an algorithm to normalize ciliary length fluctuations to controls, identify an event start point, and assign each event to one of the three categories described above (Fig 2B and S2 Fig, see Materials and methods for full description of algorithm strategy). Length curves were normalized by time (to 1,000 arbitrary units) and ciliary length (to the maximum length of each cilium) (Fig 2C–2E). The Gradual averaged curve shows early ciliary shortening with event start points distributed along the curve, followed by a period of consistent shortening in the last approximately 150 normalized time units as the slope of the curve increases (Fig 2C). The Instant averaged curve appears nearly flat until the last point; start points were nearly all clustered in the last approximately 10 normalized time units (Fig 2D). The Combined averaged curve features a period of slight slope characteristic of Gradual dynamics followed by an Instant cilia-loss event, with start points distributed along the curve (Fig 2E).

Cilia-loss events occurred throughout the entire 12-hr imaging window; hourly frequency of event start points and end points are shown in S2D–S2G Fig. These results demonstrate asynchronicity and further heterogeneity of cilia-loss behaviors. However, end points (completion of cilia loss) were most frequent between 1–3 hrs post serum addition, consistent with the enrichment of noncanonical ciliary structures observed in fixed cells (Fig 1F), further supporting that these structures are intermediates of cilia-loss events. The nature of individual loss events did not change consistently over time, suggesting that factors other than time after serum stimulation influence cilia-loss mechanism.

Ciliary-loss rates spanned several orders of magnitude (10^−3^−10^1^ μm/min), with the slowest Gradual events occurring over several hours, and the fastest Instant events (10^1^ μm/min) occurring in less than 90 seconds (Fig 2F). Within the Combined category, the rate of the first Gradual step (0.08 μm/min ± 0.26) and second Instant step (3.41 μm/min ± 2.10) were not significantly different from those of the Gradual-only (0.08 μm/min ± 0.11) and Instant-only (3.88 μm/min ± 1.80) rates, respectively (Fig 2F). Further, the majority of ciliary length (72.6 ± 4.5%) was lost during the Instant stage. Thus, Combined ciliary loss likely represents both Gradual and Instant mechanisms occurring within one loss event, rather than an independent third behavior with biphasic dynamics.

Strikingly, we found that the Instant and Combined groups together comprised 84% (*n* = 69) of the observed events (Fig 2G). Thus, events with Instant dynamics, which occur within seconds, are the predominant behavior for terminal ciliary loss in our experimental conditions. Rates of Gradual loss were heterogeneous but approximately consistent with previously reported rates of resorption [36,43,44], while Instant dynamics were significantly more rapid than would be expected for resorption. To test whether Instant loss dynamics might be consistent with what has previously been described as deciliation, we examined the dynamics of deciliation induced by dibucaine, which likely acts by raising intracellular calcium [47,49,50,61]. Dibucaine-induced deciliation had qualitative features and a dynamic profile similar to serum-induced Instant loss (S3 Fig and Fig 2A.2). Therefore, we propose that deciliation is the physical mechanism underlying Instant cilia loss.

**Fig 3:**
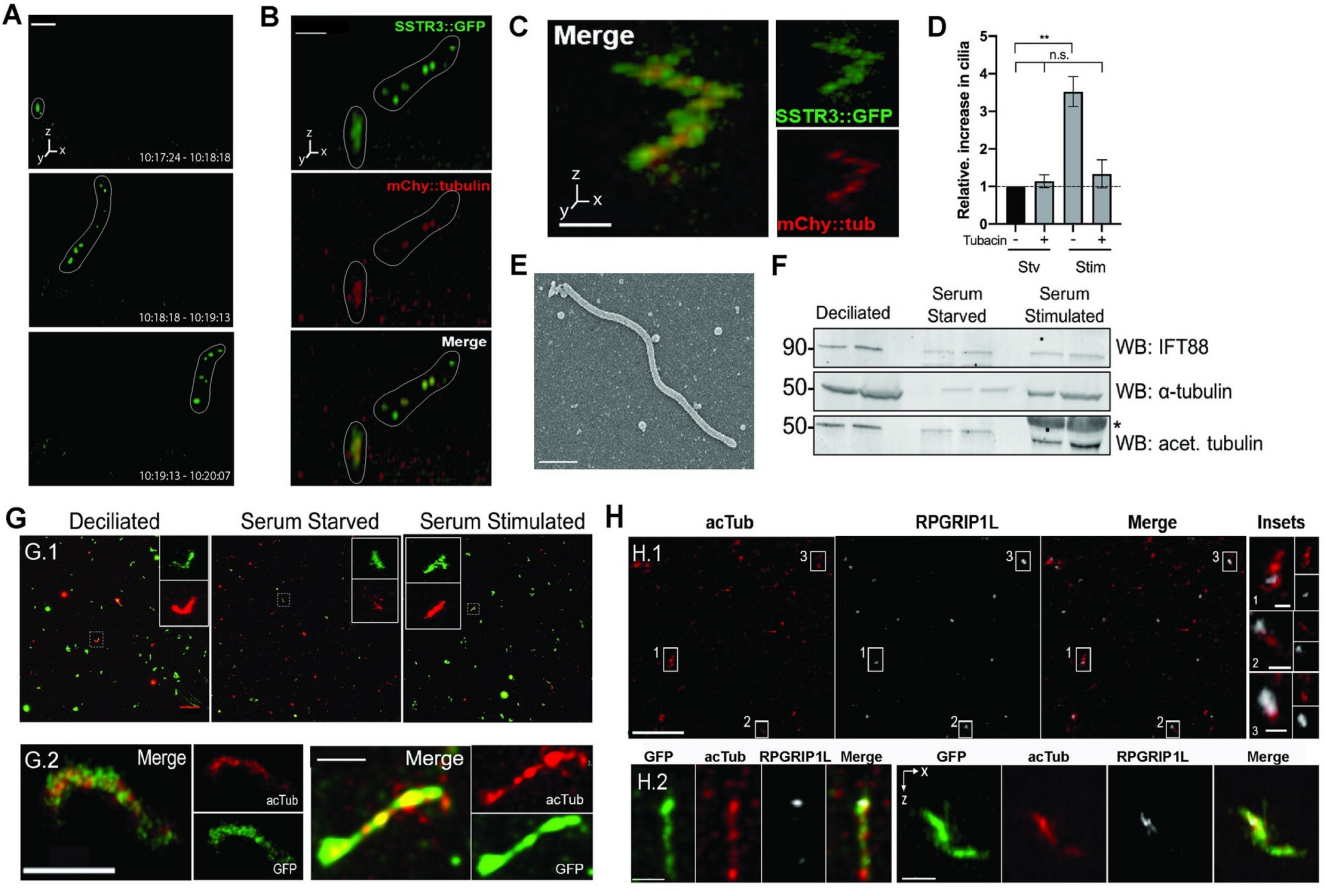
Observation and validation of whole-cilium shedding. A) Serum-stimulated cell, imaged at 54-second intervals. A short ciliary membrane (in white outline) is visibly shed from the cell surface. Time labels are intervals of stack acquisition. Scale bar = 5 μm. B) Partial ciliary shedding in IMCD3-SSTR3::GFP cells transiently expressing mCherry-α-tubulin, imaged at 30-second intervals. Intact and shed portions of the cilium are demarcated with a white outline. Tubulin is visible in shed fragments. Scale bar = 3 μm. C–E) Ciliary immune capture. Serum-stimulation media were incubated on immobilized anti-SSTR3 antibody. Isolated cilia were observed by C–D) confocal fluorescent microscopy (scale bar = 2 μm) and E) SEM. Scale bar = 500 nm. C–D) Immune-captured cilium from IMCD3-SSTR3::GFP cells transfected with mCherry-α-tubulin. D) Normalized quantification of (C), defined as elongated SSTR3+ objects at least 1.5 μm in length. Tubacin treatment reduces the prevalence of captured cilia. Cilia counts were normalized to the control serum-starved, untreated condition (-Serum -Tubacin) for each experiment, set to a value of 1 (horizontal dotted line). Data from four independent experiments, images from 10 randomly selected fields of view per sample, 63X magnification. Statistical significance determined by Welch *t* test. Source data can be found in supporting data file S3 Data. F–G) Filter-spin concentration of shed cilia. Serum-stimulation media from IMCD3 cells with no endogenous fluorescence was concentrated either by (F) centrifugation pelleting for subsequent western blot or (G) filtration centrifugation for immunofluorescence. Media from artificially deciliated cells (starved cells treated with high-calcium buffer to induce bulk deciliation) were included as a positive control. F) Western blot of concentrated material shows presence of ciliary markers at expected molecular weights: α-tubulin and acetylated tubulin. Molecular weights are indicated (kDa). Asterisk (*) denotes position of a large BSA band present in samples derived from serum-containing medium. Two replicates per condition are shown as side-by-side bands. G) Filtration-concentrated samples were processed for immunofluorescence against IFT88 and α-tubulin (G.1: top row, scale bar = 20 μm). Representative insets show full-length cilia: 6.82 μm (deciliated, 5.94μm (starved), and 6.10μm (stimulated). G.2, bottom row: High-magnification deconvolved images of individual cilia stained for GFP and acetylated tubulin. Scale bars = 2 μm. H) Filter centrifugation–concentrated cilia from deciliated controls (H.1: top row, scale bar = 10 μm; inset scale bars = 1 μm) and serum-stimulated cells (H.2: bottom row, scale bars = left: 1 μm, right: 2 μm) stained with acetylated tubulin and RPGRIP1L. BSA, bovine serum albumin; IFT88, intraflagellar transport 88; IMCD3, inner medullary collecting duct 3 cell line; SSTR3::GFP, somatostatin receptor 3::green fluorescent protein.

In a rare instance, we observed direct shedding of the entire visible cilium from the surface of a serum-stimulated cell (Fig 3A and S5 Movie). The shed cilium in the z-stack shown in Fig 3A has a fragmented appearance. Due to the rapid timescale of the shedding event, the shed cilium can travel a considerable distance (>1 μm) in the time between individual z-slices (roughly 2.6 seconds) within the stack. As a result, the shift in apparent location of the same object in subsequent slices causes the final imaged object to appear distorted—artificially elongated or with the appearance of separated fragments. We note that the segments in such sequences were visualized as a comigrating cluster, rather than dispersing independently, consistent with the interpretation that they are parts of one structure imaged at slightly different times. Therefore, we interpret the fragmented appearance of the cilium in Fig 3A as the result of motion of the imaged object during acquisition, rather than a biological change in morphology, and suggest that these images represent a deciliation event.

The rapid nature of the deciliation event shown in Fig 3A (<54 seconds) and the diffusion of the shed ciliary remnant(s) away from the site of origin is consistent with the Instant loss dynamics found in 84% of serum-induced disassembly events (Fig 2G) and in dibucaine-treated cells (S3 Fig). In addition, cilia shed under our culture conditions from cells expressing mCherry-α-tubulin contained tubulin in shed ciliary fragments (imaged at 30-second intervals, Fig 3B) suggesting that the axoneme is shed together with the ciliary membrane. These results further support the hypothesis that Instant cilium loss represents ciliary disassembly via deciliation.

### Recovery of whole cilia from culture media demonstrates that deciliation occurs during ciliary disassembly

If deciliation is a major mode of cilium disassembly, we would expect to be able to recover whole cilia from culture medium. Such recovered material should have lengths consistent with whole cilia (≥1.5 μm, see S2 Fig) and contain membrane- and axoneme-specific components. We developed two methods to enrich for ciliary fragments spontaneously released at low concentration from serum-stimulated cells (Fig 3C–3G and S4 Fig). First, we used immune-capture of fluorescently-labeled cilia to directly visualize unperturbed ciliary fragments. Culture medium from serum-stimulated IMCD3::SSTR3-GFP cells transiently expressing mCherry-α-tubulin was incubated on imaging dishes coated with an antibody against the extracellular domain of SSTR3 (S4A Fig). Samples were imaged, without fixation, by fluorescence; we could reproducibly identify cilia marked by both SSTR3-GFP and mCherry-α-tubulin captured in this manner (Fig 3C). We interpret the jagged appearance of the cilia as due to thermal motion of the sample during stack acquisition of material that is not firmly fixed to the substrate. Compared to control serum-starved medium, serum-stimulated medium yielded a 3.5-fold increase in captured cilia. In addition, pretreatment with tubacin decreased the number of captured cilia to control levels (Fig 3D). Finally, following this procedure in unlabeled IMCD3 cells, we observed objects with dimensions consistent with intact shed cilia after fixation and imaging by scanning electron microscopy, demonstrating that deciliation is not a result of SSTR3::GFP expression (Fig 3E).

The cilia immune–capture method was limited by low concentration and further sample loss due to the instability of antibody-bound cilia, which hindered subsequent compositional analysis by immunofluorescence or biochemistry. Therefore, we used a complementary method to increase yield, in which culture medium was subjected to a series of filtration and centrifugation steps to concentrate ciliary material approximately 500-fold (Fig 3F–3G and S4B Fig). As a positive control, serum-starved cells were artificially deciliated with high-calcium buffer [62] (see Materials and methods). Immunoblotting against intraflagellar transport 88 (IFT88), α-tubulin, and acetylated tubulin further confirmed enrichment of ciliary proteins in the concentrated medium (Fig 3F), and lack of DAPI-stained material indicated that isolated samples were free of large cellular debris (S4C Fig). Immunostaining for axoneme markers (IFT88, α-tubulin, and acetylated tubulin) demonstrated increased abundance of ciliary structures, many with the dimensions (1.5–7 μm) expected of whole cilia (Fig 3G).

In *Chlamydomonas*, flagellar severing can occur at two distinct sites of flagellar autotomy (SOFA): proximal and distal to the transition zone [63]. To determine the site of deciliation with respect to the transition zone in IMCD3 cells, we immunostained isolated ciliary material with an antibody against the transition zone marker RPGRIP1L. Distinct RPGRIP1L puncta asymmetrically colocalized with one end of elongated acetylated tubulin signal, indicating the presence of transition zone components in many isolated cilia (Fig 3H). Thus, deciliation can occur from the proximal transition zone, consistent with the frequent observation of complete removal of the ciliary membrane in Instant and Combined cilia loss (Fig 2A.3, 2A.4 and 2G). The fact that RPGRIP1L is not present in all acetylated tubulin–positive structures further suggests that deciliation might also occur at intermediate locations along the cilium, as observed in Fig 3B. Together, these results indicate that whole and partial cilia, including membrane and axoneme components, are shed during ciliary loss. In summary, via two independent ciliary-isolation methods, we show that deciliation occurs during serum-induced cilium loss and is likely to be the phenomenon underlying Instant loss dynamics.

### p60 katanin overexpression promotes Instant ciliary loss

We next sought to gain insight into the regulation of deciliation. A central requirement for complete deciliation is the disruption of axonemal microtubules near the ciliary base [44,51,63,64]. Although this process has been observed in several organisms [47,49,50,65], the underlying machinery and mechanism of deciliation have been difficult to elucidate. The microtubule-severing enzyme katanin has been proposed as a candidate for this function in *Chlamydomonas* and *Tetrahymena* [46,63,66,67]. Katanin has been demonstrated to localize to both proximal and distal regions of the transition zone in *Chlamydomonas* flagella [67], but *pf19* mutants, which are defective in the p60 catalytic subunit of katanin, do not have a deflagellation defect [68]. In mammalian cells, katanin has a general regulatory relationship with primary cilia [51,69]. Katanin is required for microtubule rearrangement in mitotic spindle formation [69–72], but its role in primary cilia loss is poorly understood.

**Fig 4:**
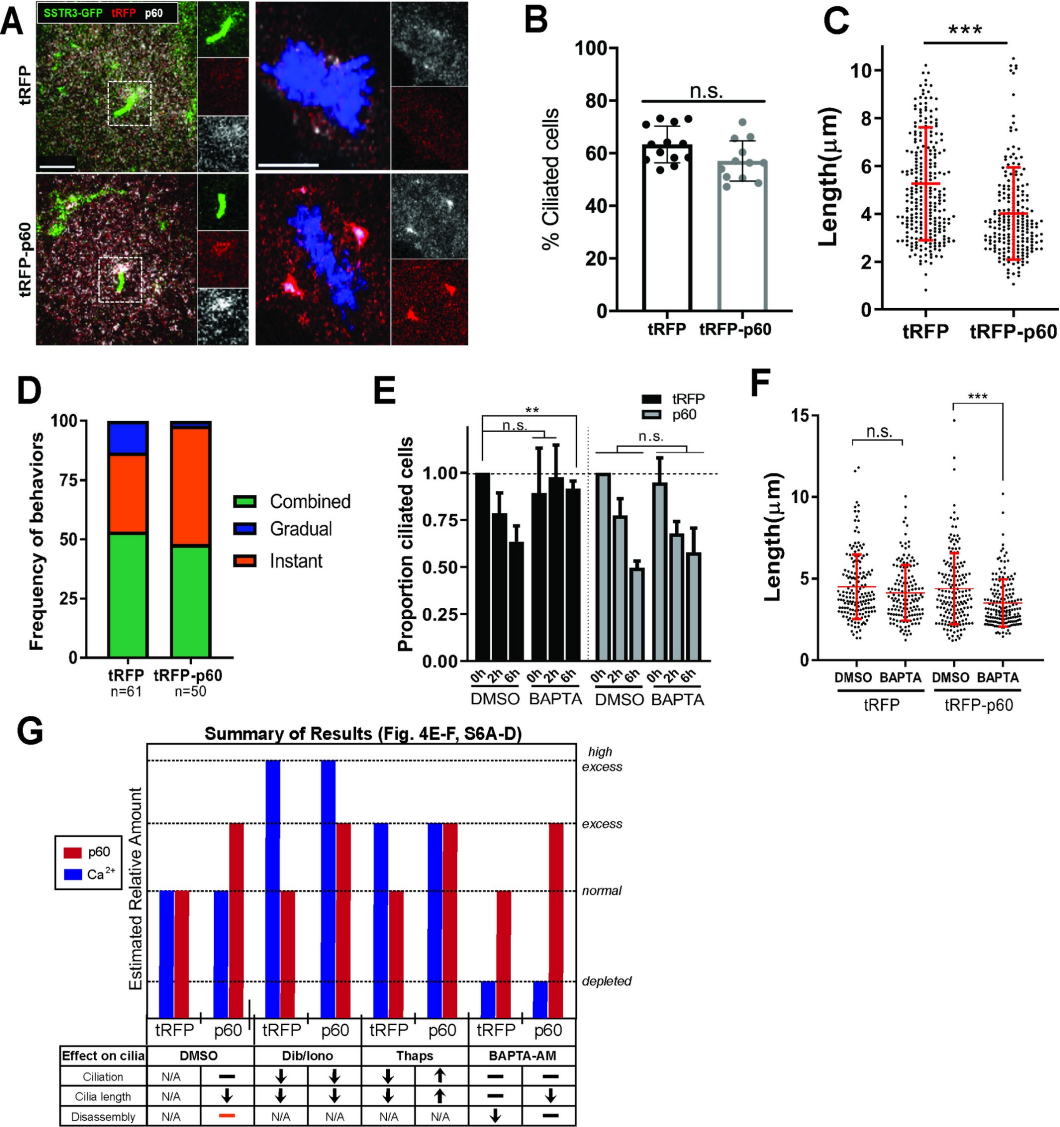
tRFP-p60 overexpression reduces ciliary length, promotes Instant loss, and counteracts effects of [Ca^2+^]_i_ modulation on cilia. A–D) IMCD3-SSTR3::GFP cells stably expressing tRFP or tRFP-p60 fusion. A) Localization of p60 to ciliary base and spindle poles in tRFP and p60 cells immunostained with a polyclonal antibody against p60 (green: SSTR3::GFP; red: tRFP; white: total p60). Scale bars = 5 μm (left) and 8 μm (right). B) Percent ciliation calculated from fixed starved tRFP and tRFP-p60 cells. Data from at least seven independent experiments. C) Cilia lengths measured from confocal stacks of live cells. Data pooled from four independent experiments (tRFP, *n* = 278; p60, *n* = 221). Statistical significance from Mann–Whitney U test. D) Relative frequencies of Gradual, Instant, and Combined dynamic behaviors in tRFP and tRFP-p60 cells. Data pooled from at least four independent experiments. E–F) Effects of BAPTA-AM on (E) ciliary abundance and disassembly. Cilia counts from fixed cells, normalized to respective starved DMSO controls. tRFP and p60 cells were serum starved and pretreated with DMSO or 1 μM BAPTA-AM (30 min) and subjected to serum stimulation and fixation at indicated time points. Data from three independent experiments, at least 100 cells counted per condition. Statistical significance was determined by unpaired *t* test. F) Cilia length measurements from live cells. BAPTA-AM data are compared to DMSO control for each cell line for statistical analysis by Mann–Whitney U test. Source data can be found in supporting data file S4 Data. G) Summary of results from Fig 4E–4F and S6 Fig. Orange bar: tRFP-p60 cells have no defect in overall ciliary disassembly but undergo Instant deciliation more frequently. We make two assumptions to approximate the relative differences in p60 and [Ca^2+^]_i_ levels between our experimental manipulations: 1) tRFP-p60 cells have “excess” p60 compared to “normal” levels in tRFP control cells, and 2) thapsigargin may produce a [Ca^2+^]_i_ that is physiological but higher than the baseline state (labeled “excess”), whereas dibucaine and ionomycin induce “high excess” levels of [Ca^2+^]_i_. [Ca2+]i, intracellular calcium concentration; IMCD3, inner medullary collecting duct 3 cell line; SSTR3::GFP, somatostatin receptor 3::green fluorescent protein; tRFP, turbo red fluorescent protein.

To examine the potential for regulation of ciliary loss by katanin, we manipulated katanin level by overexpression. We generated IMCD3 cells stably coexpressing SSTR3-GFP and tRFP-p60 or turbo red fluorescent protein (tRFP) as a control (S5A and S5B Fig). Consistent with previous reports [46,68,72–75], we observed diffuse localization of p60 in the cytoplasm and mitotic spindle poles in both tRFP- and tRFP-p60–expressing cells (Fig 4A and S5A Fig). In addition, in approximately 30% of ciliated cells, p60 localized near the base of the primary cilium (Fig 4A). Cytoplasmic acetylated tubulin intensity was reduced in serum-starved and serum-stimulated tRFP-p60 cells compared to the control by immunofluorescence and western blot (S5C–S5F Fig), consistent with increased severing and destabilization of microtubules caused by activity of katanin overexpression [65,73,76].

Next, we determined whether tRFP-p60 katanin expression influenced overall assembly and loss of cilia. Total levels of ciliation in response to serum starvation (Fig 4B) and stimulation (S5C Fig) were unaffected; however, ciliary length was significantly reduced in tRFP-p60 cells (Fig 4C). Furthermore, inhibition of ciliary loss by tubacin and cytochalasin D was unaffected in tRFP-p60 cells (S5G Fig). Therefore, tRFP-p60 katanin expression influences ciliary structure but is not sufficient to induce ciliary disassembly in serum-starved cells, and any effect of p60 overexpression likely occurs downstream of HDAC6 activity.

We next asked whether tRFP-p60 katanin expression affects ciliary loss dynamics in serum-stimulated cells. In cells expressing only tRFP, terminal cilium loss by Instant dynamics comprised 86.7% of disassembly events (*n* = 61, 33.3% Instant and 53.3% Combined), consistent with our analysis in parental cell lines in Fig 2G. However, in tRFP-p60–overexpressing cells, Gradual dynamics were virtually eliminated, while the frequency of Instant loss increased, resulting in 98% of events featuring Instant terminal cilium loss (*n* = 50, 50% Instant and 48% Combined) (Fig 4D). Thus, tRFP-p60 expression shifts the distribution of disassembly behaviors toward Instant ciliary loss dynamics. These results suggest that increased katanin activity is capable of modulating ciliary disassembly behavior by promoting deciliation with Instant dynamics and that the distribution of heterogeneous disassembly behaviors is tunable by mechanistic regulators.

### Katanin p60 overexpression blocks the effects of [Ca^2+^]_i_ modulation on ciliary disassembly

High-intracellular calcium concentration ([Ca^2+^]_i_) triggers deciliation, and many methods of experimentally inducing deciliation do so by raising [Ca^2+^]_i_ ([46,47,49–51,62,77–82], also see Fig 3F, Fig 3G and S3 Fig). p60 katanin function and [Ca^2+^]_i_ are both necessary for experimentally induced severing of isolated flagellar–basal body complexes in vitro [46]. Additionally, calcium-calmodulin signaling functions upstream of Aurora A kinase, a key regulator of ciliary loss upstream of HDAC6 [23,35,83], suggesting a broader role for cytoplasmic calcium in regulating ciliary loss via all of the behaviors we have identified. Thus, we hypothesized that calcium and katanin functions may cooperate in serum-induced ciliary loss. We asked whether, in the presence of high [Ca^2+^]_i,_ the frequency of cilium loss would be exacerbated by expression of tRFP-p60. Cells were serum starved to promote ciliation and then treated with small molecule drugs to modulate [Ca^2+^]_i_ levels. In both tRFP- and tRFP-p60–expressing cells, raising [Ca^2+^]_i_ with either 1 μM ionomycin, an ionophore [84], or 190 μM dibucaine reduced ciliary abundance and length to a similar degree (S6A and S6B Fig). Treating cells with 5 μM thapsigargin, which raises [Ca^2+^]_i_ by releasing intracellular stores [50,85], also reduced ciliation but not cilia length [50] in control tRFP-expressing cells. Intriguingly, this effect was reversed in tRFP-p60–expressing cells, which exhibited a slight increase in ciliation and cilia length in response to thapsigargin (S5C and S5D Fig). Overall, tRFP-p60–expressing cells did not exhibit elevated rates of ciliary loss induced by high [Ca^2+^]_i_.

Conversely, we asked what effects depletion of [Ca2+]_i_ would have on ciliary disassembly in tRFP- and tRFP-p60–expressing cells. BAPTA-AM, a cell-permeable Ca^2+^ chelator [86], did not affect ciliary length in starved cells but inhibited ciliary loss in serum-stimulated tRFP cells (Fig 4E and 4F), consistent with published work [23]. However, cilium loss was not impaired in serum-stimulated tRFP-p60 cells (Fig 4E), and ciliary length in starved cells was reduced compared to DMSO-treated tRFP-p60 cells (Fig 4F). Therefore, tRFP-p60 katanin expression can overcome the requirement for [Ca^2+^]_i_ in ciliary loss. We summarize these results in a schematic in Fig 4G. Overall, we found that overexpression of tRFP-p60 eliminated or reversed the effects of both increasing [Ca^2+^]_i_ with thapsigargin (S6C and S6D Fig) and reducing [Ca^2+^]_i_ with BAPTA-AM (Fig 4E and 4F). Taken together, these results indicate that [Ca^2+^]_i_ and katanin do not act cooperatively to promote ciliary loss. Rather, the negative regulation of cilia by [Ca^2+^]_i_ appears to be mitigated or reversed in the presence of excess p60 katanin.

## Discussion

We characterized serum-induced ciliary-loss dynamics and behaviors of primary cilia, a process critical to tissue homeostasis and development in vertebrates. Here, we present a model for ciliary loss based on these results and discuss implications of that model.

**Fig 5:**
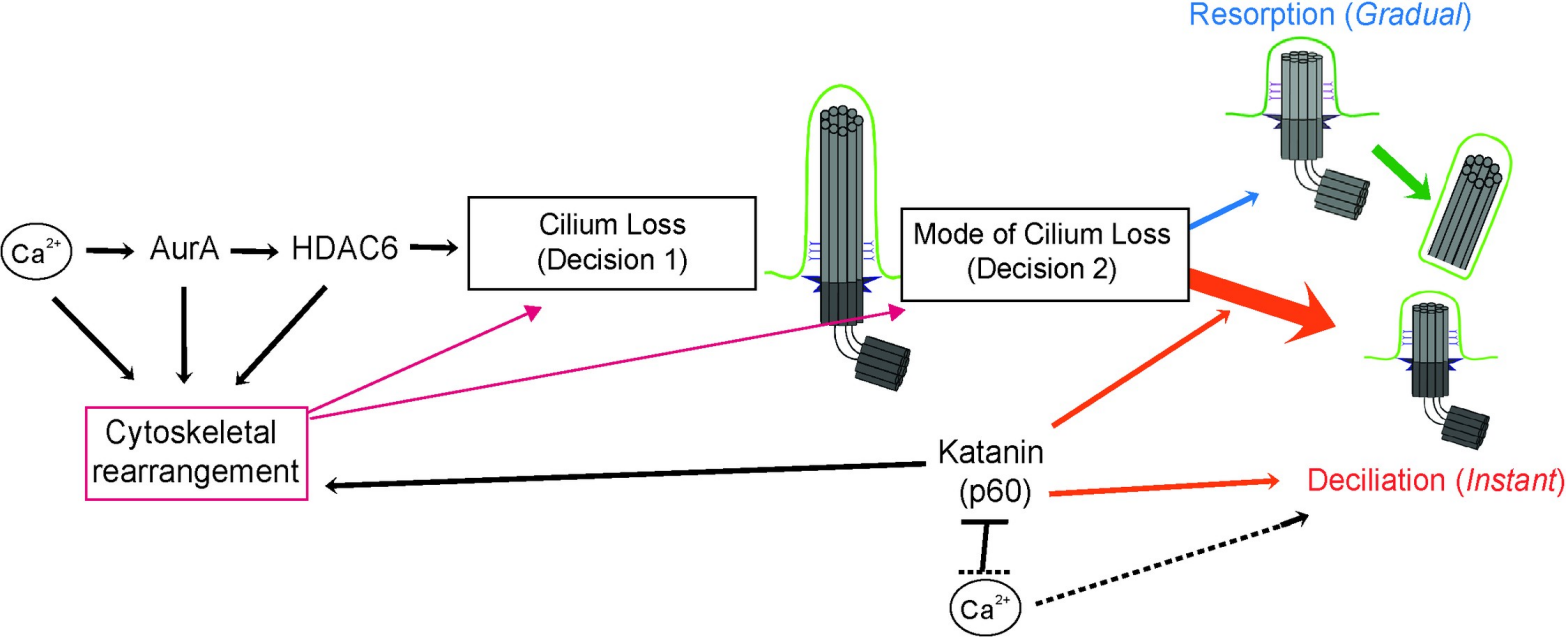
Model for regulation of ciliary disassembly. Major regulators of ciliary disassembly function at two decision points: whether (Decision 1) and how (Decision 2) to disassemble the cilium, which is dominated by deciliation (orange) but also features resorption (blue) and Combined resorption-deciliation (green). Katanin (p60) and Ca^2+^ can promote deciliation at Decision 2 independently and/or antagonistically. Regulators of disassembly may further contribute to either decision indirectly via modulation of cytoskeletal rearrangement (magenta). See text for full description.

Our results suggest that there are at least two mechanisms of ciliary loss in mammalian cells: resorption, in which the axoneme is depolymerized and ciliary contents are incorporated into the cell, and deciliation, in which the axoneme is excised near its base. We have incorporated these in a model (Fig 5) in which ciliary loss requires at least two major decision points: decision 1, when to remove the cilium and decision 2, when to invoke Instant deciliation. Decision 1 is controlled by Aurora A, HDAC6, and other elements [24,34,54,87,88]. Decision 2, in IMCD3 cells, results in deciliation immediately (Instant), after a delay (Combined), or never (Gradual). We assume that resorption of the cilium, as the initial behavior in Combined dynamics, is the default mode of cilium loss and that rapid deciliation, when invoked, overrides the slower-acting resorption. Thus, cilium loss is a tunable decision in at least two ways: when to remove the cilium and the mechanism by which that removal is accomplished, both of which are likely to differ in different cell types and different contexts.

In most cases of Instant cilium loss, the entire visible cilium was lost within seconds; however, in some cases, detachment of the distal portion of the cilium could be observed (Fig 3A). In addition, we observed the presence of the transition zone protein RPGRIP1L in some cilia isolated from cell culture medium. This suggests that ciliary detachment can occur at multiple sites along the cilium. In *Chlamydomonas*, there are two known SOFAs [63], directly above and below the transition zone, and katanin has been shown to localize to both sites via immunogold electron microscopy [67]. Flagellar severing at one site or the other seems to be associated with functional context—distal severing is associated with stress response, and proximal severing occurs during mitotic flagellar disassembly [63]. Whether these deciliation sites translate to a functional difference in the mammalian primary cilium is not understood. For instance, it could be that distal severing, which leaves the transition zone intact, may allow for rapid regeneration of the cilium, while proximal severing may serve as a “terminal deciliation” in a cell cycle context in which regrowth is suppressed; indeed, this could be a third decision that a cell undertakes during cilia loss.

To better understand how deciliation is regulated in in IMCD3 cells, we manipulated p60 katanin and intracellular calcium levels and found that the activity of overexpressed p60 katanin biases cilium-loss events nearly exclusively toward deciliation, likely representing an intervention at Decision 2. Whether and how katanin might sever axoneme microtubules in deciliation, or whether it is indeed required for deciliation [68], remain critical outstanding questions. Katanin may also indirectly influence cilia, perhaps by severing centrosome-associated microtubules and interfering with ciliary protein trafficking (consistent with the observed diffuse staining pattern at the cilium base, Fig 4A) or by modulating the available pool of cytoplasmic tubulin [89,90]. These hypotheses could help explain why tRFP-p60 expression negatively affects cilia length but not formation in untreated cells.

Calcium is necessary for ciliary loss [23] and is sufficient to drive deciliation [47,50,79,91], thus acting at both Decisions 1 and 2. Although the role of calcium is likely multifaceted, it seems that calcium and increased p60 katanin activity may function independently and also negatively interact at the Decision 2 nexus (Fig 4E–4F, S6C and S6D Fig). Despite the requirement for Ca^2+^ for disassembly (Decision 1) and deciliation (Decision 2) in normal conditions, tRFP-p60 cells were able to undergo ciliary loss after [Ca^2+^]_i_ chelation, likely via deciliation. This suggests the existence of a Ca^2+^-independent deciliation pathway, as has been suggested previously [50]. Alternatively, overexpressed p60 may promote cilium loss via resorption in the absence of [Ca^2+^]_i_. This relationship may be consistent with the finding that calcium binding inactivates p60 severing activity in vitro [92]. We speculate that the activities of katanin and [Ca^2+^]_i_ in modulating ciliary behavior may depend on their relative levels in the cell (Fig 4G).

What is the significance of the multiple mechanisms of ciliary loss coexisting in the same cells? In *Chlamydomonas*, the anterograde IFT kinesin mutant *fla-10*, which normally undergoes passive flagellar shortening due to IFT imbalance [43], switches to deflagellation in the presence of Ca^2+^ at the restrictive temperature. Conversely, deflagellation-incompetent mutants (*fa1*, *fa2*, *adf*) undergo flagellar resorption in response to acid shock, which normally induces deflagellation [93]. This work is directly in line with our two-decision model, suggesting that once an internal or external cue to disassemble the cilium/flagellum is detected (Decision 1), the cell will “find a way” to remove its cilium—if the preferred or default mode of disassembly (Decision 2) is unavailable or unfavorable, an alternative mechanism can be used. The recent development of an engineered inhibitable KIF3A/3B recapitulated the *fla-10* phenotype of in NIH3T3 cells—inhibition of motor activity induced cilium loss. Intriguingly, the consistent reduction in cilia number over 8 hrs was accompanied by only a minor reduction in cilium length, suggesting that cilia were lost by rapid deciliation [94]. Future studies could take advantage of this tool to examine the dynamic behaviors of cilia during this process, as well whether modulation of p60 katanin, intracellular calcium levels, and other potential regulators influence cilia-loss dynamics induced by a cell cycle–independent stimulus.

Ciliary-loss behaviors varied both in the dynamics (10^−3^ to 10^1^ μm/min) and physical process (resorption versus deciliation). The coexistence of resorption and deciliation in the same cilium (Combined dynamics) is intriguing and may suggest independent or differential regulation of the distal and proximal portions of the cilium [36]. It might be, for example, that structural features of the axoneme, such as doublet–singlet microtubule interface or post-translational modifications, contribute to differential regulation of ciliary regions. We were unable to directly and specifically observe the axoneme in our live-cell imaging experiments. However, in fixed serum-stimulated cells, we identified several noncanonical structures that hint at the fate of the axoneme during ciliary loss. These included discontinuous axoneme staining that might reflect partial breaks away from the base, short axoneme stubs that could represent a remnant of a severed or resorbed cilium [95,96], and even axonemes without corresponding ciliary membrane staining, possibly representing a portion of axoneme retracted into the cell, as has been previously reported [39]. While these interpretations are speculative due to the markers used and the nature of static representations of this dynamic process, the relatively low abundance of these structures in starved and tubacin-treated conditions indicates that they may represent ciliary loss intermediates.

We emphasize that loss of the cilium involves a complex interplay between general ciliary trafficking and regulation, cytoskeletal dynamics, and intracellular signaling ([4,9] Fig 5) and that our manipulations of calcium and katanin are best viewed as initial probing into the molecular nature of these decisions. Further work is required to understand the molecular mechanisms and must take into account that many key regulators of ciliary loss have additional roles in cytoskeletal regulation [22,32,54] and other cellular functions, including calcium [97,98], AurA [99,100], HDAC6 [101–104], and katanin [70,71,105].

We observed deciliation as a means for cell cycle–linked ciliary loss, whereas previous descriptions of such behavior in mammalian cells have only been under conditions of experimentally induced toxicity and stress [50,52]. The morphology and composition of isolated cilia confirmed two major points supporting the interpretation that the observed Instant loss events represent deciliation: 1) the entire ciliary membrane can be shed from cells as an intact structure, and 2) shed ciliary membranes contain tubulin, suggesting that the axoneme is severed and shed along with the ciliary membrane. Interestingly, a related phenomenon—the release of a small membrane segment from the ciliary tip, referred to as apical abscission, decapitation, or release of ciliary ectosomes—has been described in several contexts [88,106,107], but it is unclear whether axoneme components are present in these structures. Ciliary decapitation was previously reported as an initiating step in the cilia-loss process [88] as well as in assembling cilia [9]. It would be particularly interesting to test whether there is a mechanistic link between these events by investigating whether PI(4,5)P_2_– and F-actin–mediated membrane constriction that drives decapitation participates in deciliation from the ciliary base as well as other locations along the cilium. Finally, the implications of our findings for understanding cilia loss should be considered in the context of the general features of cultured kidney-derived epithelial cells, in comparison to and contrast with cilia in other experimental approaches, cell types, tissues, and organisms. Although most cilia share a similar core structure and associated machinery, differences in structure and function (i.e., primary versus motile/specialized), and the relative size of cilia to the cell body, are likely to be relevant to the mechanisms of ciliary assembly and loss.

## Materials and methods

### Cell culture

IMCD3 cells were grown in DMEM-F12 medium with 10% fetal bovine serum and 1% penicillin-streptomycin-kanamycin antibiotic cocktail at normoxic conditions. Cells were passaged every 2–3 days at a dilution of 1:10–1:20. Cells were tested for mycoplasma with Sigma LookOut Mycoplasma PCR Detection Kit (Cat# MP0035) as directed by the manufacturer, and incidences of mycoplasma contamination were treated with Mycoplasma Removal Agent (MP Biomedicals, #093050044). Following decontamination, experiments that were potentially affected by mycoplasma contamination were repeated at least three times to determine any difference in results, and no significant differences were observed.

### Serum starvation and stimulation

Cells were seeded in 24- or 6-well dishes with glass coverslips for imaging following fixation or 35 mm glass-bottomed MatTek dishes (#P35G-0-10-C) for live imaging. Cells were seeded in 24-well dishes at a density of 1.5 x 10^4^ cells and 6-well and 35 mm MatTek dishes at 1–1.5 x 10^5^ to achieve 50%–70% confluence next day. For serum starvation, cells were washed once with 0.2% DMEM-F12 + PSK, then grown in 0.2% DMEM-F12 + PSK for 24 hrs. Serum stimulation was by either readdition of FBS directly to dishes to 10% final concentration or replacement with 10% FBS DMEM-F12.

### Antibodies

The following antibodies and dilutions were used: acetylated tubulin mouse monoclonal 6-11B-1 (1:1,000 for IF and WB) (Sigma-Aldrich Cat# T7451); pericentrin rabbit polyclonal Poly19237 (1:500 for IF) (Covance Cat #PRB-432C, now BioLegend); Arl13b rabbit polyclonal (1:250–1:500 for IF) (Proteintech Cat# 17711-1-AP); N19-SSTR3 antibody (rabbit polyclonal) (Santa Cruz Cat #sc-11610, discontinued); IFT88 rabbit (1:500 for IF and WB) (GeneTex, Cat#79169); α-tubulin YL1/2 (1:1,000 for IF and WB), (ThermoFisher #MA1-080017); alpha-tubulin DM1a (1:1,000 for IF and WB) (ThermoFisher #62204); rabbit monoclonal anti-p60 EPR5071, (1:250 IF), (Abcam Cat# ab111881); rabbit polyclonal anti-KATNA1, (1:100–250 IF) (Proteintech Cat#17560-1-AP), anti-RPGRIP1L (Proteintech 55160-1-AP). Anti-rabbit GFP (1:250) (Life Technologies, #A11122); anti-mouse GFP (1:1,000) (Roche, #11063100). Secondary antibodies used were anti-mouse Rhodamine (1:1,000) (Jackson ImmunoResearch, #715-295-150); anti-rabbit FITC (1:1,000) (Jackson ImmunoResearch, #111-095-003); anti-rabbit Alexa647 (1:200) (Life Technologies, #A21245); anti-mouse Alexa647 (1:200) (Life Technologies, #A21236); Hoescht (1:1,000–2,000) (Molecular Probes, #H-3570).

### Chemicals

Tubacin (Sigma-Aldrich, #SML0065) at 2 μM in DMSO; dibucaine hydrochloride (Sigma-Aldrich #285552) at 190 μM in DMSO. The following were used at 1 μM in DMSO: Cytochalasin D (Sigma-Aldrich #PHZ1063), Thapsigargin, (Sigma-Aldrich #T9033). BAPTA-AM (Sigma-Aldrich #A1076). Ionomycin 10-mM stock was a gift from the Rich Lewis Laboratory, Stanford University.

### Generation of stable cell lines

IMCD3-SSTR3::GFP-mCherry::PACT: mCherry::PACT was cloned from a pLV plasmid (pTS3488, created by multisite Gateway cloning by Christian Hoerner) onto a pLV-Puro-EF1a construct using Gibson cloning. Lentivirus with the cloned construct was generated in HEK293T and used to infect IMCD3-SSTR3::GFP (gift from Nachury laboratory, [55]) under selection with 800 ng/μL puromycin for 4–5 days. Infected cells were FACS sorted into polyclonal populations by mCherry fluorescence intensity, and a pool of low-expressing cells was selected to prevent overexpression phenotypes of a centrosomal protein.

#### Katanin expression constructs

Mammalian expression constructs for turboRFP and turboRFP::p60 (mKATNA1 [NM_011835.2], build 37.2, Gene ID: 23924) were designed and ordered from VectorBuilder. All constructs were amplified by transformation in DH5α and maxi-prep (Qiagen #12165). IMCD3-SSTR3::GFP cells were transfected with each construct with ThermoFisher Lipofectamine 3000 according to manufacturer's protocol (#L3000015). The next day, cells were subjected to G418 selection (800 ng/μL for 5–6 days). Cells were sorted by FACS into low-, medium-, and high-expressing pools and maintained in DMEM-F12 10% FBS + PSK and 250 ng/μL G418 to maintain transgene expression.

### Transient transfection

mCherry-α-tubulin mammalian expression construct (gift from Angela Barth) was transfected into IMCD3-SSTR3::GFP cells. Transfections were performed using Lipofectamine 3000 transfection reagent according to manufacturer’s protocol.

### Immunofluorescence microscopy

Generally, fixation for immunofluorescence microscopy was done with 100% methanol for 5 minutes at −20°C, followed by washes with 0.1% Triton X-100 in PBS at room temperature for 2 minutes, and washed three times in PBS. Samples were blocked for 1 hr at RT or overnight at 4°C in 2% BSA, 1% goat serum, 75 mM NaN_3_. Antibodies were diluted to the indicated concentrations in blocking buffer. Primary antibody incubations were performed for 1 hr at RT or overnight at 4°C. Secondary antibody incubations were performed for 1–2 hrs at RT. Following each antibody incubation, samples were washed three times in PBS + 0.05% Tween-20 for 5 minutes each at RT.

Images were acquired with a Zeiss Axiovert 200 inverted epifluorescence microscope and a 63x objective, or a Leica SP8 scanning laser confocal microscope with LASX Software, using mercury or argon lamps with white light laser excitation, and a 63x 1.4 NA objective. Exposure times were constant during each experiment. For imaging of serum-starved and serum-stimulated cells, fields of view were selected based on DAPI staining by two criteria: 1) to select for moderate cell density, in order to avoid effects of high density on cell cycle and ciliation and 2) to eliminate bias in percent cilia quantifications from scanning by ciliary markers.

### Live-cell confocal microscopy

Cells were cultured on glass-bottomed Mattek dishes and imaged in DMEM-F12 media with 15 mM HEPES without phenol red. Movies were acquired 4–12 hrs after serum stimulation with a Leica SP8 scanning laser confocal microscope using 0.5-μm z-slices, 30–90-second intervals, autofocus (Best Focus function), in a 37°C incubator, and red and green channels were acquired simultaneously. The video file was saved as .lif from LASX software and opened in Imaris x64 8.0.2 as a 3D render for analysis of cilia disassembly dynamics and basal body positioning.

### Data analysis

Cilia counts and length measurements were performed either manually in Fiji or Imaris x64 8.0.2 and 9.2.1 or through semiautomated detection in Imaris. Manual analysis involved detecting ciliary membrane, marked by an enrichment of SSTR3::GFP above background threshold, that were adjacent to a centriole (mCherry::PACT in dual-fluorescent cells or pericentrin immunofluorescence in single [SSTR3::GFP-expressing] or nonfluorescent cells), to distinguish from accumulations of SSTR3+ membrane elsewhere in the cell. Manual length measurements in Fiji [108] were made with the line function, and in Imaris (Bitplane, version 8.0.2 and up) with the Measurement tool. Generally, single z-plane images were analyzed in Fiji or Imaris, while confocal z-stacks were analyzed in Imaris which allowed more accurate length measurement due to the 3D render (Surpass) capability. When possible, length measurements in confocal images were semi-automated in Imaris using the Surfaces function to create an artificial object encompassing the ciliary membrane, and exporting Bounding Box data as a proxy for length (the longest dimension of the object).

For live cell serum stimulation experiments, movies were visually scanned in Imaris for examples of disassembling cilia. Images of each disassembling cilium were cropped by time (from t0 to several mins after complete loss) and position (restricted to area of occupancy during the that time window) and then saved in a separate file. To generate ciliary length curves, the ciliary membrane was isolated as an artificial object using the Surface function. When possible, the object was automatically tracked over consecutive time points with length data generated at each time point. In cases where automatic tracking was not possible due to low signal-to-noise of ciliary membrane fluorescence, measurements were taken manually at 15–30 minute intervals until the initiation of ciliary disassembly, and at each time point during the disassembly event.

#### Matlab

Raw length measurement data from disassembling cilia movies were compiled into an Excel spreadsheet. The data were imported into a Matlab algorithm which performed Gaussian smoothing using a moving average and normalization to starved control cilia dataset. The start point of the cilium loss event was defined by scanning backwards from the final point (time at which length = 0 μm) until the derivative (length difference between consecutive time points) is not different in magnitude than the mean control slope (−0.005 μm/min). Based on this start point, each cilium loss event was categorized as follows: as follows: Gradual if the start point was earlier than the penultimate time point (T_final-1_); Instant if the length at the penultimate time point was above 1.532 μm (threshold based on measurement error); and Combined if both criteria are true. An output file containing disassembly rates, start and end time, start and end length, and proportion length lost per disassembly stage was generated for all cilium loss events. Full annotated script is available on Github: https://github.com/mmirvis/Mirvis-et-al.-2019-PLOS-Biol.

### Cilia isolation

Cell culture: clones of IMCD3 cells, either unsorted and stably expressing GFP-SSTR3 or FACS-sorted for medium expression of GFP-SSTR3, were grown on 15-cm dishes at 3 x 10^6^ cells/dish in DMEM/F12 with 10% FBS and antibiotics for 24 hrs. The cells were washed 3x with HDF wash buffer (136 mM NaCl, 5 mM KCl, 5 mM glucose, 4 mM NaHCO_3_, 0.7 mM EDTA), and medium was replaced with DMEM/F12 and 0.2% FBS and antibiotics (serum-starved) for 24 hrs. Then, all dishes were washed 3x with HDF buffer and half received phenol-red free DMEM/F12 with 0.2% FBS (serum-starved), and the other half received phenol red free DMEM/F12 with 10% FBS (serum-stimulated) for 24 hrs. Total serum starved time was 48 hrs and total serum stimulated time was 24 hrs.

#### Immune-capture method

Preparation of antibody-immobilized imaging dishes: glass in 35-mm glass-bottomed imaging dishes (MatTek) was functionalized by plasma cleaning at 250 moor, low setting, 45–60 seconds. Dishes were silanized with 500 μL of 2.5% triethoxysilyl-undecanal (TESU) in 100% ethanol, covered with Parafilm and incubated at RT for 1hr. Dishes were washed 3x with 100% ethanol, then baked at 85°C for 3 hrs. Next, silanized dishes were treated with the following series of reagents for 1hr at RT unless stated otherwise, with three PBS washes in between steps: 1) 50 mM NHS-LC- LC-biotin in water, 2) 5mg/ml neutravidin for 1hr at RT, 3) 300 μg/ml biotin-Protein A 4) block with 15 mM D-biotin in DMSO for 30 minutes, 5) anti-rabbit SSTR3 N19 (extracellular N-terminus) antibody (100–200 μg/mL) at 37°C, followed by one PBS wash. These protocols adapted from Dr. Nicholas Borghi [109].

#### Sample preparation

Culture medium was collected and subjected to centrifugation for 10 minutes at 1,000 x g at 4°C to remove large cell debris. Samples were then kept on ice until plating on treated dishes or stored at 4°C for a maximum of 1 day. 4 mL serum-stimulated or -starved medium was incubated on a treated MatTek dish overnight at 4°C, followed by three gentle PBS washes. Samples were then imaged directly, without fixation with a Leica SP8 confocal microscope.

#### SEM

Antibody-immobilized MatTek dishes incubated with serum stimulation medium were fixed for SEM in 4% PFA, 2% glutaraldehyde, and 0.1M Na cacodylate. Glass bottoms were removed, processed for imaging, and imaged with a Hitachi S-3400N VP SEM scope in the Beckman Imaging Facility, Stanford University.

#### Filter-spin concentration method

Harvest of cilia: Deciliation of starved IMCD3 cells (positive control): Serum-stimulated or -starved culture medium, or fresh culture medium (with 10% FBS, an additional control) was removed from six 150-cm dishes, combined, and centrifuged at 1,000 x g at 4°C for 10 minutes in the A-4-81 rotor, Eppendorf 5810R centrifuge to remove large cellular debris. Cells were washed two times with warm PBS containing 0.4% EDTA. 10 mL was added to a MatTek dish and incubated for 10 minutes at 37°C, followed by gentle up and down pipetting to remove cells from dish. An aliquot of cell suspension was removed for cell count. Cells were centrifuged at 13,000 x g for 5 minutes at RT. The cell pellet was resuspended in 5 mL ice cold deciliation buffer (62) (112 mM NaCl, 3.4 mM KCl,10 mM CaCl2, 2.4 mM NaHcO3, 2 mM HEPES, pH7.0, and a protease inhibitor tablet [Roche]). The cell suspension was incubated at 4°C for 15 minutes with end-over-end rotation, and then centrifuged at 1,000 x g for 5 mins at 4°C in an Eppendorf centrifuge. The resulting supernatant was used for biochemistry and immunostaining.

#### Biochemistry

Half of the supernatant material from deciliated, serum-starved or -stimulated cells was centrifuged at 21,000 x g for 15 minutes in JA25.5 rotor in Beckman Coulter Avanti J-25I centrifuge at 4°C. The supernatant was carefully removed, and pellets were resuspended in 160 μl of sample buffer (1% SDS, 10 mM Tris-HCl, pH 7.5, 2 mM EDTA). Samples were boiled at 95°C for 8 mins, and equal volumes were separated by 10% PAGE and transferred to PVDF. Blots were blocked (2% BSA, 1% normal donkey and goat serum in TBS, pH 7.4) for 1 hr at RT or overnight at 4°C. Membranes were blotted with YL1/2 (1:1,000), mouse acetylated-tubulin antibody (1:1,000), and IFT88 rabbit antibody (1:500) in blocking buffer for 1 hr at RT. Blots were washed 5x with TBST. Secondary anti-rabbit, anti-mouse, or anti-rat antibodies labeled with either IRDye680CW or IRDye800CW (Li-Cor Biosciences, #926–32213), at 1:30,000 dilution were incubated with blots for 30 minutes at RT. Blots were washed five times with TBST and scanned on Licor Odyssey scanner (Li-Cor BioSciences).

#### Immunofluorescence

Half of the supernatant material from deciliated, serum-starved, and serum-stimulated cells was concentrated using a 250 ml 0.2 μm PES filter unit with low-power house vacuum to reduce the volume (20 or 40 mL from one or two 15-cm dishes, respectively) to 2 mL, and finally a Millipore Ultrafree-MC filter (PVDF 0.2 μm size #UFC30GV100) to reduce the volume to approximately 0.5 mL. 5 μl of concentrated supernatant was pipetted onto an acid-treated glass slide. A 22-mm acid-treated circular glass coverslip was placed on the sample, and the slide was immediately plunged into liquid nitrogen for approximately 5 seconds. After removing the slide, the coverslip was removed and fixed in −20°C 100% methanol for 5 minutes. Alternatively, 400 μm of the sample were loaded into an 8-well glass-bottomed LabTek dish (Sigma-Aldrich, # Z734853), and centrifuged at 3,500 x g for 20 minutes, 4°C. Immunofluorescence staining was performed as described above.

### Statistics

All analyses were performed in GraphPad Prism. Statistical tests used for each analysis are indicated in the figure legends. No explicit power analysis was used to determine sample size. All experiments were performed with at least three biological replicates, i.e., samples from independent cell culture passages. When used, technical replicates (i.e., repeats from the same cell culture passage) were averaged for each biological replicate. In brief, comparisons of mean values such as mean percent cilia across replicate experiments were compared using an unpaired *t* test. Analyses of individual measurements such as cilia length were subjected to normality tests (Kolmogorov–Smirnoff, D’Agostino and Pearson, and Shapiro–Wilk). If data passed all normality tests, unpaired *t* test was used; if not, the Mann–Whitney U test was used. If data passed normality by some tests but not others, both types of analyses were performed. Results were similar between parametric and nonparamentric tests unless stated otherwise.

## Supporting information

S1 Fig Characterization of immunostained serum-stimulated cells. A) Serum-starved and stimulated cells were immunostained for PCNT to mark the basal body (white) and acTub to mark the axoneme (red). Scale bar = 10 μm. Nuclei and cell boundaries are outlined in yellow. B) Serum stimulation is accompanied by an increase in mitotic cells, inhibited by tubacin treatment. Quantifications are based on means of three independent experiments, with 150–200 cells analyzed per condition per replicate. Error bars = SEM. Source data can be found in supplementary data file S1 Data. acTub, acetylated tubulin; PCNT, pericentrin.
(TIF)

S2 Fig Schematized workflow for automated analysis of ciliary disassembly dynamics. A) 10 control nondisassembling cilia from starved cells were imaged at 90-second intervals over a 12-hr period and analyzed. Cumulative metrics are shown here (μm). Slight changes in length and stochastic length fluctuations are inherently present, and used as a baseline for analysis of disassembling cilia. Marked in yellow—maximum standard deviation of length is used as a proxy for length measurement error and is thus used as a threshold length for Instant disassembly; average slope of best fit line represents background decline in length over 12-hr imaging period. B) Matlab workflow. Scale bar = 5 μm. Raw data are run through a smoothing function. Derivatives are calculated, then normalized to background reduction (A) to identify start point of disassembly event. Lastly, disassembly behaviors (Gradual, Instant, and Combined) are assigned as described in the text and Materials and methods. Source data can be found in supplementary data file S2 Data. Matlab script available at https://github.com/mmirvis/Mirvis-et-al.-2019-PLOS-Biol.
(TIF)

S3 Fig Dynamics of dibucaine-induced ciliary shedding are consistent with serum-induced Instant disassembly. Starved cells were treated with 190 μm dibucaine and imaged by confocal microscopy at 30-second intervals. A) Still images from a representative ciliary shedding show complete ciliary loss in under 30 seconds. B) Length measurements from A show Instant disassembly dynamics. Source data can be found in supplementary data file S2 Data.
(TIF)

S4 Fig Schematics of cilia capture methods. A) Immune capture of shed cilia. Medium from serum stimulated cells is incubated on an imaging dish bearing immobilized antibody against the SSTR3 membrane marker. B–C) Filter-spin concentration of shed cilia. B) Medium from serum-stimulated cells is concentrated either by centrifugation pelleting for subsequent western blot or by vacuum and centrifugation filtration for immunofluorescence. Medium from cells subjected to artificial deciliation (serum-starved cells treated with high calcium buffer) was included as a positive control. C) Visualization of concentrated cilia, showing native SSTR3::GFP fluorescence, immunofluorescence against acetylated tubulin, and DAPI staining. Scale bar = 20 μm. SSTR3, somatostatin receptor 3
(TIF)

S5 Fig tRFP-p60 overexpression reduces cytoplasmic acTub intensity but does not impair overall serum-induced ciliary disassembly. A) Localization of p60 in tRFP- and tRFP-p60–overexpressing cells SSTR3::GFP native fluorescence (green), turboRFP native fluorescence (red), total p60 immunofluorescence (white), and merge with DAPI staining. Scale bar = 20 μm. B) Quantification of total p60 immunofluorescence intensity from maximum intensity projections of confocal z-stacks. Data from three independent experiments, 30–50 cells per experiment. Statistical significance calculated by unpaired *t* test. C) Starved and 2-hr–stimulated tRFP and tRFP-p60 cells were fixed and stained for acTub (red, center column). Scale bar = 20 μm. Insets show acTub channel alone. Inset scale bar = 10 μm. D) Quantification of A). Mean cellular acTub intensity was normalized to cellular tRFP intensity. Data pooled from three independent experiments, 80–100 cells per condition. Statistical significance from Mann–Whitney U test. E) Western blot against α-tubulin (DM1α, top green) and acTub (bottom, red). F) Quantification of band intensity of (E), normalized to tRFP. Three independent experiments, statistical significance by unpaired *t* test. G) Serum-starved cells pretreated with DMSO, 2 μM Tubacin, or 1μM CytoD were serum stimulated (6 hrs). Proportion of ciliated cells was calculated by normalizing to Starved + DMSO for each cell line (dotted line). *N* = 3 experiments. Source data can be found in supplementary data file S4 Data. acTub, acetylated tubulin; SSTR3, somatostatin receptor 3; tRFP, turbo red fluorescent protein.
(TIF)

S6 Fig tRFP and tRFP-p60 cells exhibit similar responses to elevated [Ca2+]i by ionomycin and dibucaine but not to thapsigargin. tRFP and tRFP-p60 cells were starved and pretreated with DMSO, A–B) 190 μM dibucaine (30 m), 1 μM ionomycin (30 minutes), and C–D) 5 μM Thps (1 hr). All experiments, including BAPTA-AM studies in Fig 4, were performed in parallel and use the same values for DMSO controls. A and C) Cilia counts are from fixed populations and normalized to respective DMSO controls. Statistical significance was determined by unpaired *t* test. B and D) Length measurements from confocal z-stacks of live cells, taken in three dimensions using Imaris. Statistical significance was determined by Mann–Whitney U test for non-normal distribution. Data from at least three independent experiments. Source data can be found in supplementary data file S4 Data. Thps, thapsigargin; tRFP, turbo red fluorescent protein.
(TIF)

S1 Movie Primary cilium of a serum-starved cell. Imaged at 90-second intervals over 12 hrs, 45 fps. The cilium undergoes rapid length fluctuations and a slight overall reduction in length at 0.003 μm/min. See Fig 2A.1.
(MP4)

S2 Movie Primary cilium loss by Gradual dynamics. Imaged at 90-second intervals, 45 fps. Cilium disassembles with an overall rate of 0.016 μm/min. See Fig 2A.2.
(MP4)

S3 Movie Primary cilium loss by Instant dynamics. Imaged at 90-second intervals, 15 fps. Cilium is lost in under 90 seconds, approximate minimum rate of 4.72 μm/min. See Fig 2A.3.
(MP4)

S4 Movie Primary cilium loss by Combined dynamics. Imaged at 46-second intervals, 25 fps. Cilium undergoes Gradual shortening (0.029 μm/min), followed by Instant loss (>5.69 μm/min). See Fig 2A.4.
(MP4)

S5 Movie Primary cilium shedding. Imaged at 54 second intervals. Ciliary membrane is released from cell surface (27:12–29:00). See Fig 3A.
(MP4)

S6 Movie Primary cilium shedding. Top view of S5 Video.
(MP4)

S1 Data Mean percentages of ciliated cells, mitotic cells, and non-canonical ciliary structures in response to serum stimulation in fixed IMCD3 cells. Data corresponding with Fig 1 and S1B Fig.
(XLSX)

S2 Data Analysis of live cell ciliary disassembly dynamics, including raw data from individual cilia, and results of MATLAB analysis. Data corresponding with Fig 2, S2 Fig and S3B Fig.
(XLSX)

S3 Data Quantification of cilia isolated by immune capture. Data corresponding with Fig 3D.
(XLSX)

S4 Data Effects of katanin and calcium modulation on ciliary structure and behavior, including characterization of tRFP-p60 overexpression and ciliary disassembly dynamics, and results of calcium drug experiments. Data corresponding with Fig 4, S5 Fig and S6 Fig.
(XLSX)

## Abbreviations

[Ca2+]_i_: intracellular calcium concentration
IFT88: intraflagellar transport 88
IMCD3: inner medullary collecting duct 3
HDAC6: histone deacetylase 6
PACT: pericentrin-AKAP450 centrosome targeting domain
PCNT: pericentrin
SOFA: sites of flagellar autotomy
SSTR3::GFP: somatostatin receptor 3::green fluorescent protein
tRFP: turbo red fluorescent protein
