# Supplementary figures and images for: Primary cilium loss in mammalian cells occurs predominantly by whole-cilium shedding
Source: PLoS Biol. 2019 Jul 17;17(7):e3000381. doi: 10.1371/journal.pbio.3000381 (PMC6699714; doi:10.1371/journal.pbio.3000381)

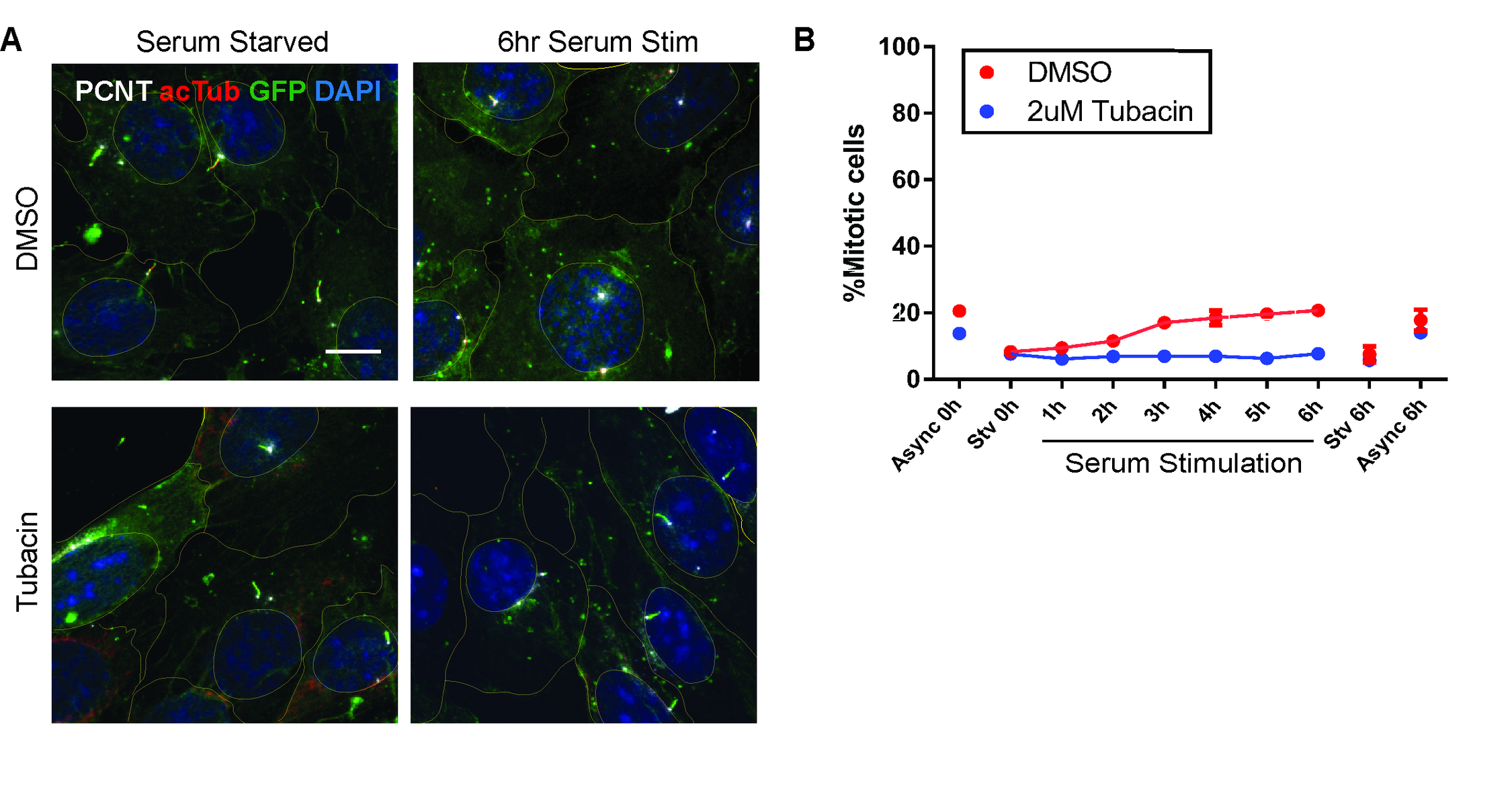

Supplement: S1 Fig — A) Serum-starved and stimulated cells were immunostained for PCNT to mark the basal body (white) and acTub to mark the axoneme (red). Scale bar = 10 μm. Nuclei and cell boundaries are outlined in yellow. B) Serum stimulation is accompanied by an increase in mitotic cells, inhibited by tubacin treatment. Quantifications are based on means of three independent experiments, with 150–200 cells analyzed per condition per replicate. Error bars = SEM. Source data can be found in supplementary data file S1 Data. acTub, acetylated tubulin; PCNT, pericentrin. (TIF) [file pbio.3000381.s001.tif]

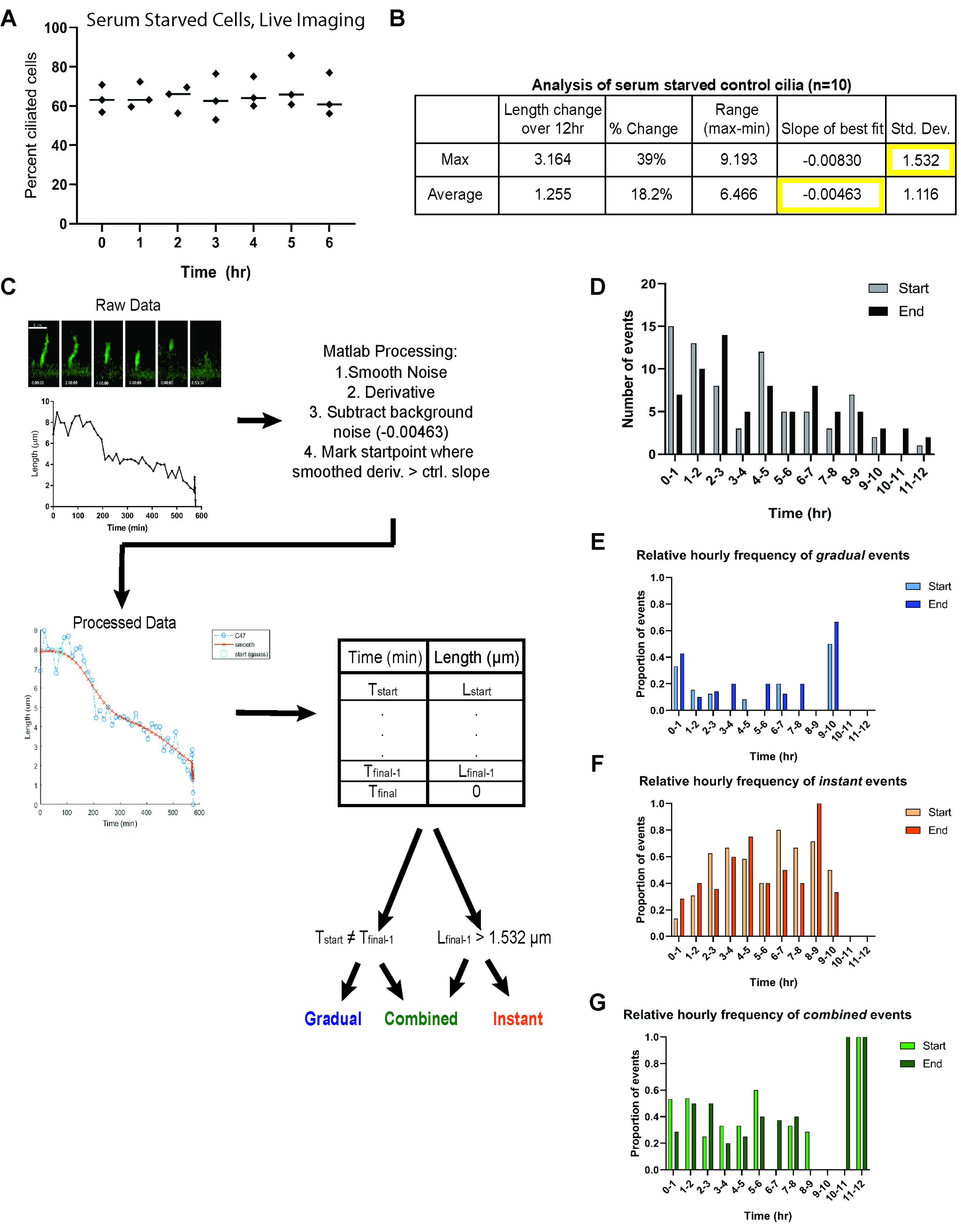

Supplement: S2 Fig — A) 10 control nondisassembling cilia from starved cells were imaged at 90-second intervals over a 12-hr period and analyzed. Cumulative metrics are shown here (μm). Slight changes in length and stochastic length fluctuations are inherently present, and used as a baseline for analysis of disassembling cilia. Marked in yellow—maximum standard deviation of length is used as a proxy for length measurement error and is thus used as a threshold length for Instant disassembly; average slope of best fit line represents background decline in length over 12-hr imaging period. B) Matlab workflow. Scale bar = 5 μm. Raw data are run through a smoothing function. Derivatives are calculated, then normalized to background reduction (A) to identify start point of disassembly event. Lastly, disassembly behaviors (Gradual, Instant, and Combined) are assigned as described in the text and Materials and methods. Source data can be found in supplementary data file S2 Data. Matlab script available at https://github.com/mmirvis/Mirvis-et-al.-2019-PLOS-Biol. (TIF) [file pbio.3000381.s002.tif]

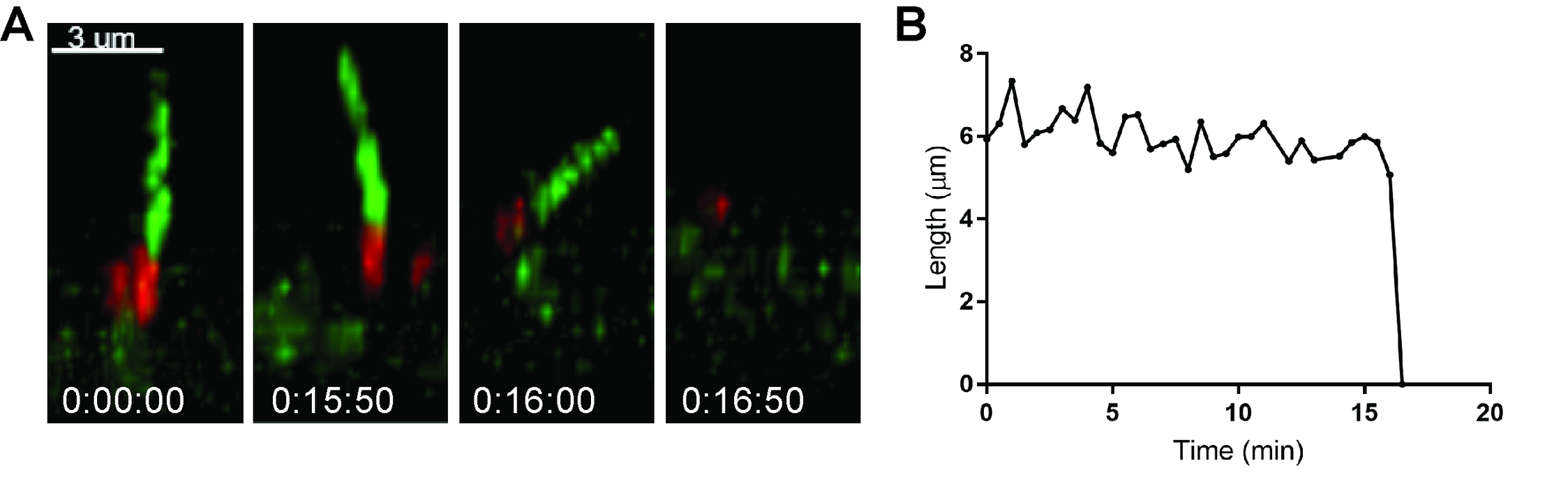

Supplement: S3 Fig — Starved cells were treated with 190 μm dibucaine and imaged by confocal microscopy at 30-second intervals. A) Still images from a representative ciliary shedding show complete ciliary loss in under 30 seconds. B) Length measurements from A show Instant disassembly dynamics. Source data can be found in supplementary data file S2 Data. (TIF) [file pbio.3000381.s003.tif]

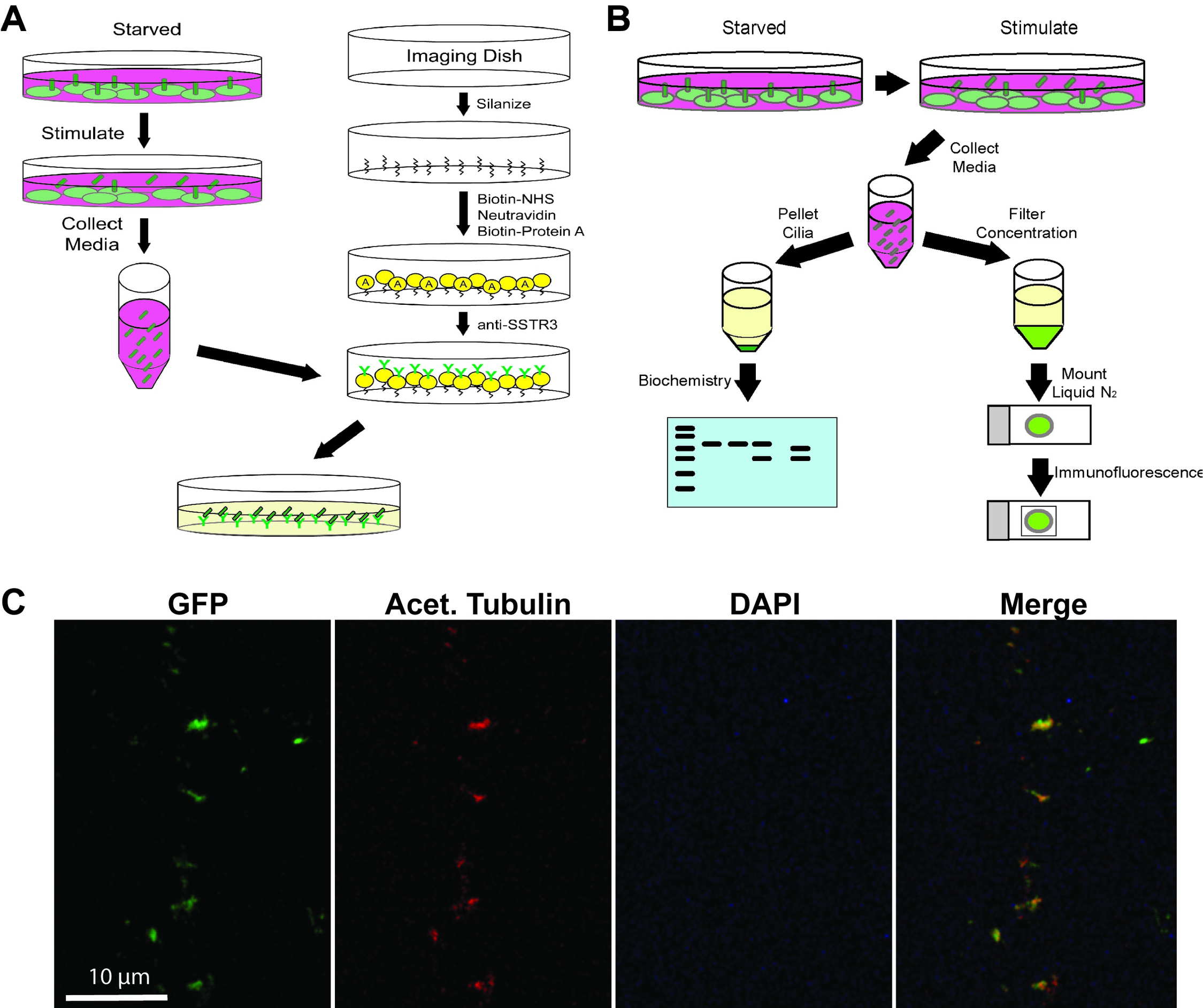

Supplement: S4 Fig — A) Immune capture of shed cilia. Medium from serum stimulated cells is incubated on an imaging dish bearing immobilized antibody against the SSTR3 membrane marker. B–C) Filter-spin concentration of shed cilia. B) Medium from serum-stimulated cells is concentrated either by centrifugation pelleting for subsequent western blot or by vacuum and centrifugation filtration for immunofluorescence. Medium from cells subjected to artificial deciliation (serum-starved cells treated with high calcium buffer) was included as a positive control. C) Visualization of concentrated cilia, showing native SSTR3::GFP fluorescence, immunofluorescence against acetylated tubulin, and DAPI staining. Scale bar = 20 μm. SSTR3, somatostatin receptor 3 (TIF) [file pbio.3000381.s004.tif]

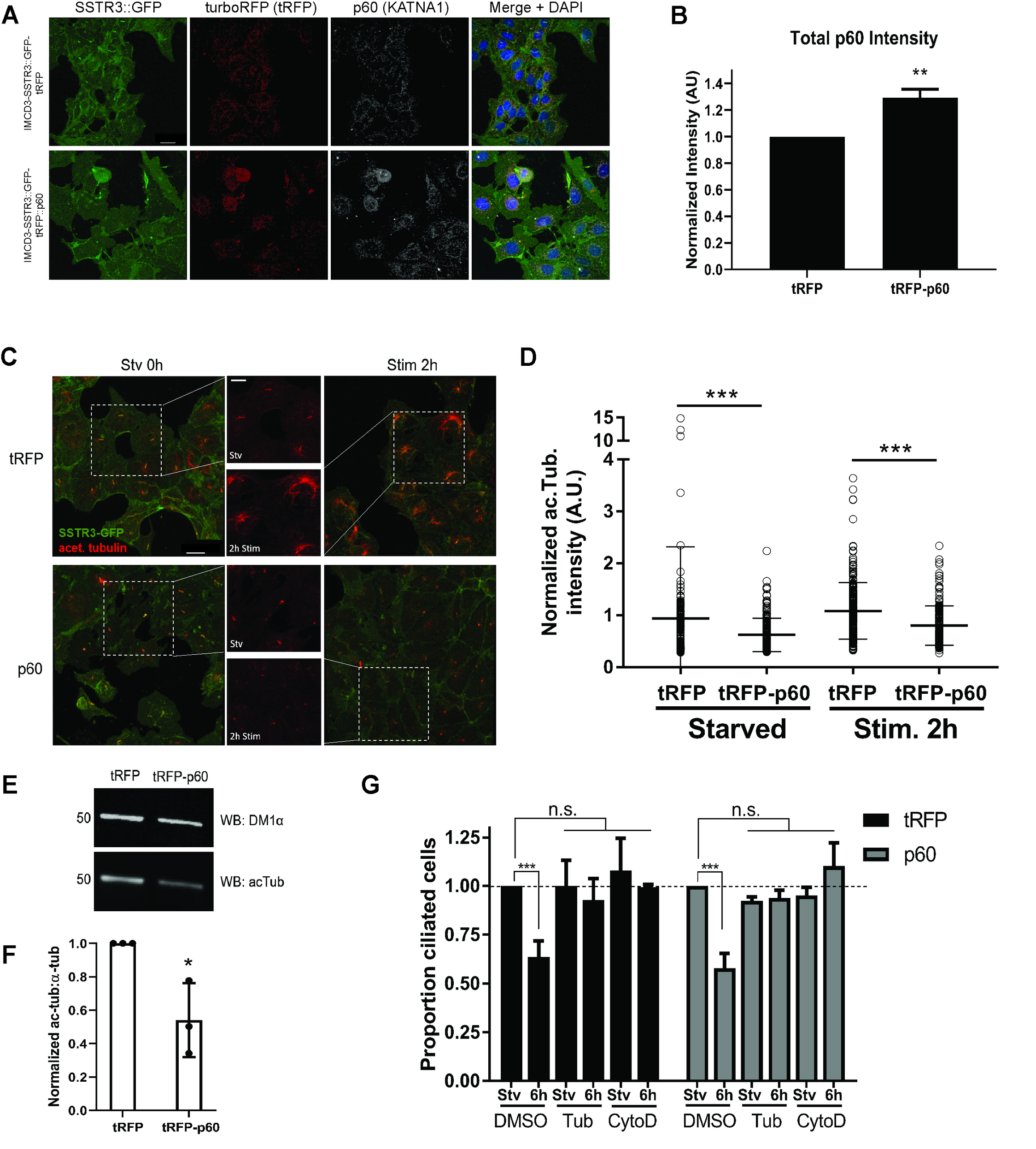

Supplement: S5 Fig — A) Localization of p60 in tRFP- and tRFP-p60–overexpressing cells SSTR3::GFP native fluorescence (green), turboRFP native fluorescence (red), total p60 immunofluorescence (white), and merge with DAPI staining. Scale bar = 20 μm. B) Quantification of total p60 immunofluorescence intensity from maximum intensity projections of confocal z-stacks. Data from three independent experiments, 30–50 cells per experiment. Statistical significance calculated by unpaired t test. C) Starved and 2-hr–stimulated tRFP and tRFP-p60 cells were fixed and stained for acTub (red, center column). Scale bar = 20 μm. Insets show acTub channel alone. Inset scale bar = 10 μm. D) Quantification of A). Mean cellular acTub intensity was normalized to cellular tRFP intensity. Data pooled from three independent experiments, 80–100 cells per condition. Statistical significance from Mann–Whitney U test. E) Western blot against α-tubulin (DM1α, top green) and acTub (bottom, red). F) Quantification of band intensity of (E), normalized to tRFP. Three independent experiments, statistical significance by unpaired t test. G) Serum-starved cells pretreated with DMSO, 2 μM Tubacin, or 1μM CytoD were serum stimulated (6 hrs). Proportion of ciliated cells was calculated by normalizing to Starved + DMSO for each cell line (dotted line). N = 3 experiments. Source data can be found in supplementary data file S4 Data. acTub, acetylated tubulin; SSTR3, somatostatin receptor 3; tRFP, turbo red fluorescent protein. (TIF) [file pbio.3000381.s005.tif]

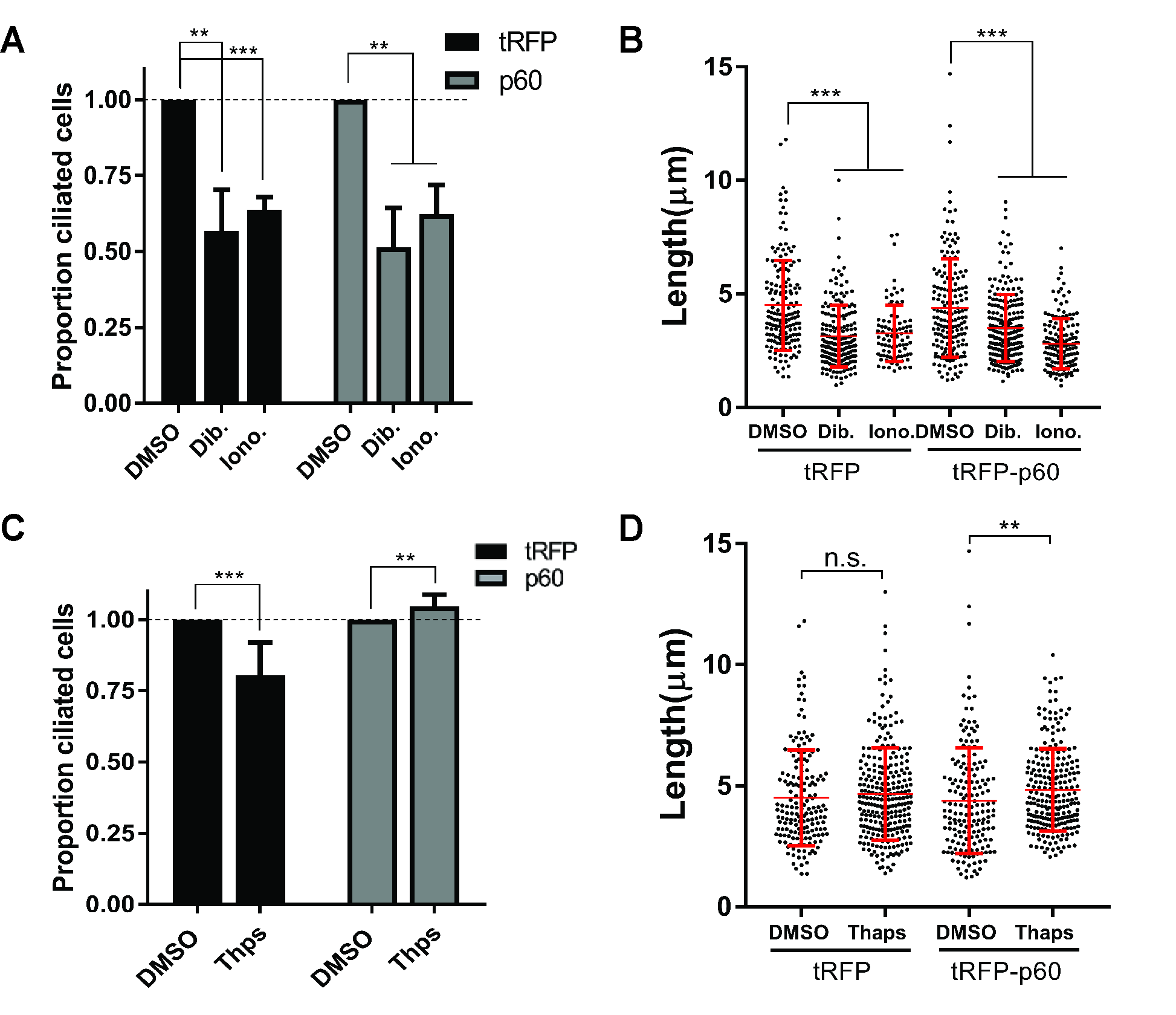

Supplement: S6 Fig — tRFP and tRFP-p60 cells were starved and pretreated with DMSO, A–B) 190 μM dibucaine (30 m), 1 μM ionomycin (30 minutes), and C–D) 5 μM Thps (1 hr). All experiments, including BAPTA-AM studies in Fig 4, were performed in parallel and use the same values for DMSO controls. A and C) Cilia counts are from fixed populations and normalized to respective DMSO controls. Statistical significance was determined by unpaired t test. B and D) Length measurements from confocal z-stacks of live cells, taken in three dimensions using Imaris. Statistical significance was determined by Mann–Whitney U test for non-normal distribution. Data from at least three independent experiments. Source data can be found in supplementary data file S4 Data. Thps, thapsigargin; tRFP, turbo red fluorescent protein. (TIF) [file pbio.3000381.s006.tif]
